# Avian Reovirus Protein p17 Functions as a Nucleoporin Tpr Suppressor Leading to Activation of p53, p21 and PTEN and Inactivation of PI3K/AKT/mTOR and ERK Signaling Pathways

**DOI:** 10.1371/journal.pone.0133699

**Published:** 2015-08-05

**Authors:** Wei-Ru Huang, Hung-Chuan Chiu, Tsai-Ling Liao, Kuo-Pin Chuang, Wing-Ling Shih, Hung-Jen Liu

**Affiliations:** 1 Institute of Molecular Biology, National Chung Hsing University, Taichung, 402, Taiwan; 2 Department of Medical Research, Taichung Veterans General Hospital, Taichung, 402, Taiwan; 3 Graduate Institute of Animal Vaccine Technology, National Pingtung University of Science and Technology, Pingtung, 912, Taiwan; 4 Department of Biological Science and Technology, National Pingtung University of Science and Technology, Pingtung, 912, Taiwan; 5 Agricultural Biotechnology Center, National Chung Hsing University, Taichung, 402, Taiwan; 6 Rong Hsing Research Center for Translational Medicine, National Chung Hsing University, Taichung, 402, Taiwan; Georgia Regents University, UNITED STATES

## Abstract

Avian reovirus (ARV) protein p17 has been shown to regulate cell cycle and autophagy by activation of p53/PTEN pathway; nevertheless, it is still unclear how p53 and PTEN are activated by p17. Here, we report for the first time that p17 functions as a nucleoporin Tpr suppressor that leads to p53 nuclear accumulation and consequently activates p53, p21, and PTEN. The nuclear localization signal (119IAAKRGRQLD128) of p17 has been identified for Tpr binding. This study has shown that Tpr suppression occurs by p17 interacting with Tpr and by reducing the transcription level of Tpr, which together inhibit Tpr function. In addition to upregulation of PTEN by activation of p53 pathway, this study also suggests that ARV protein p17 acts as a positive regulator of PTEN. ARV p17 stabilizes PTEN by stimulating phosphorylation of cytoplasmic PTEN and by elevating Rak-PTEN association to prevent it from E3 ligase NEDD4-1 targeting. To activate PTEN, p17 is able to promote β-arrestin-mediated PTEN translocation from the cytoplasm to the plasma membrane via a Rock-1-dependent manner. The accumulation of p53 in the nucleus induces the PTEN- and p21-mediated downregulation of cyclin D1 and CDK4. Furthermore, Tpr and CDK4 knockdown increased virus production in contrast to depletion of p53, PTEN, and LC3 reducing virus yield. Taken together, our data suggest that p17-mediated Tpr suppression positively regulates p53, PTEN, and p21 and negatively regulates PI3K/AKT/mTOR and ERK signaling pathways, both of which are beneficial for virus replication.

## Introduction

Nuclear pore complex (NPC), consisting of a conserved set of 30 different nucleoporin proteins, forms a channel that is the important mediator of exchange between the nucleus and cytoplasm in eukaryotic cells [1, 2]. The nucleoporin Tpr (translocated promoter region) is a 267 KDa protein that is an integral component of the NPC localized within the nuclear basket of the NPC and plays a crucial role in nucleocyplasmic transport as a scaffolding element [3–5]. It has also been demonstrated to be an oncogenic activator of the met and trk proto-oncogenes [6]. Tpr also has been uncovered to interact with Mad1 and dynein to promote proper chromosome segregation during mitosis [7]. It is required for normal spindle assembly checkpoint (SAC) response by stabilizing Mad1 ans Mad2 before mitosis [8]. Previous studies have suggested that Tpr depletion increases p53 and p21 nuclear accumulation and facilitates autophagy [9] and induces mitotic catastrophe and enhances the rate of tetraploidy and polyploidy [10]. The central domain of Tpr binds to and sequesters extra Aurora A to safeguard bipolarity [10].

In terms of molecular mechanisms, p53, phosphatase and tensin homolog deleted on chromosome 10 (PTEN), and retinoblastoma (Rb) tumor suppressors are all important gatekeepers for oncogene-induced transformation [11–13]. Working as a transcription factor, p53 can induce apoptosis, cellular senescence, and cellular quiescence [14–17]. Rb is one of targets of the cyclin dependent kinase (CDK) complexes, mainly CDK4-cyclin D, and its function depends on its interaction with E2F [18]. Phosphorylation of Rb by CDKs neutralizes its cell cycle inhibitory properties, allowing progression of G1 to S phase. An earlier study has demonstrated that p21 causes Rb dephosphorylation, which is inactivated in proliferating cells through phosphorylation by CDK2 and CDK4/6, all of which are suppressed by p21 [19]. The PTEN plays a crucial role in AKT dephosphorylation/ inactivation, thereby inducing G1 cell cycle arrest, apoptosis, and autophagy, along with the regulation of cell adhesion, migration, and differentiation [20, 21]. The PTEN is a lipid and protein phosphatase blocking phosphoinositide 3-kinase (PI3K)-dependent signaling by dephosphorylating phosphatidylinositol 3, 4, 5-trisphosphate (PIP3). The downstream molecule of PI3K, mammalian target of rapamycin (mTOR), plays a critical role in many signaling pathways which promote tumorgenesis through the coordinated phosphorylation of its target proteins directly mediating protein synthesis, cell cycle progression, cell growth, and proliferation [22–24]. Akt-mediated phosphorylation can downregulate tuberin’s GTPase-activating potential toward Rheb, which modulates mTOR complex 1 through FKBP38 by directly binding to mTOR [25].

Avian reovirus (ARV) is an important poultry pathogen which causes several diseases, including viral arthritis, chronic respiratory diseases, and malabsorption syndrome [26, 27]. Although the mortality is low, the economic losses due to the removal of birds from premature flocks are substantial. ARVs have 10 double-stranded RNA (dsRNA) genome segments which are divided into L (large), M (medium), and S (small) groups, based on their electrophoretic mobility. These genome segments encode at least eight structural and four nonstructural proteins. A previous report has indicated that ARV S1 genome segment contains three open reading frames that are translated into p10, p17, and σC proteins, respectively [28]. Earlier studies on the biological function of p10 protein have suggested that it displays membrane destabilization activity [29–31]. The σC protein has been demonstrated to be a cell attachment protein [32] and is capable of inducing cell apoptosis [23–35]. Recently, a report by Costas et al. demonstrated that p17 is a CRM1-independent nucleocytoplasmic shuttling protein [36] that shuttles between the nucleus and the cytoplasm to affect signaling pathways that regulate host cell translation, cell cycle, and autophagosome formation, paving a way for virus productive infection [37–39]. However, the precise mechanisms by which p17 modulates host factors and upstream signaling pathways remain largely unknown. In this work, we have undertaken a comprehensive investigation to explore the molecular mechanisms underlying p17-modulated host factors and upstream signaling pathways in both immortalized chicken embryo fibroblast (DF-1) and African green monkey kidney (Vero) cells. This is the first report to suggest that p17-mediated suppression of Tpr leads to p53 and p21 nuclear accumulation, which thereby activates p53, p21, and PTEN, followed by a down-regulation of PI3K/AKT/mTOR and ERK signaling pathways. These molecular events lead to cellular translation shutoff in the host cell cycle arrest and autophagosome formation, benefiting virus replication.

## Materials and Methods

### Cells and Viruses

Both DF-1 and Vero cells were maintained as previously described [39]. Cells were seeded in 6-cm cell culture dishes with 1 x 10^6^ cells 1 day before each experiment in a 37°C incubator with 5% CO_2_. All cells were cultivated in serum-free medium for 2 hours and then refreshed with the medium containing 5% FBS until cell confluence reached about 75%. The ARV s1133 strain was used in this study.

### Reagents and Antibodies

The PI3K inhibitor LY294002 and rapamycin were purchased from Merck Co. (Darmstadt, Germany). ATM-kinase-specific inhibitor caffeine and 3-methyladenine (3-MA) was purchased from Sigma-Aldrich Co. (St. Louis, USA). Y-27632 and TBB were from Calbiochem Co. (San Diego, USA). The monoclonal antibody against p17 protein of ARV is from our laboratory stock. Mouse anti-Rak, rabbit anti-NEDD4-1, mouse anti-β-arrestin, rabbit anti-nucleoporin Tpr, mouse anti-ubiquitin, mouse anti-CDK 4, mouse-anti-GSK3α/β, rabbit-anti-cyclin D1, rabbit-anti-cyclin E, rabbit-anti-cyclin A, rabbit-anti-cyclin B1, rabbit anti-E2F-1, and rabbit anti-p-p21 (T145) antibodies were purchased from Santa Cruz Biotechnology (Dallas, USA). Rabbit anti-Rock-1, rabbit anti-PI3k p85, rabbit anti-p-PI3K p85 (Tyr458)/p55 (Tyr199), rabbit anti-mTOR, rabbit anti-FoxO1, rabbit anti-FoxO3a, rabbit anti-p-FoxO1 (T24)/FoxO3a (T32), rabbit anti-p-mTOR S2448, rabbit anti-p-PTEN (S380/T382/T383), rabbit anti-PTEN, rabbit anti-p-PDK1 (S241), rabbit anti-PDK1, rabbit anti-p-AKT (T308), rabbit anti-p-AKT (S473), rabbit anti-AKT, rabbit anti-p-GSK3α (S21), rabbit anti-p-GSK3β (S9), rabbit anti-p-eIF4E (S209), rabbit anti-eIF4E, rabbit anti-p-p70S6k (T389), rabbit anti-70S6k, rabbit anti-4EBP1, rabbit anti-p-ERK (T202/T204), rabbit anti-ERK, rabbit anti-p-ATM (Ser1981), rabbit anti-ATM, rabbit anti-p-p53 (S15), mouse anti-p53, rabbit anti-histone H2A, rabbit anti-p-Rb (S780), mouse anti-Rb, rabbit anti-p21, and rabbit anti-Na/K-ATPase were from Cell Signaling (Danvers, USA). Mouse anti-β-actin antibody was from Millipore (Billerica, USA).

### Construction of the Flag-Tagged p17 and the N-Terminally and C-Terminally Truncated Flag-Tagged p17 Coding Sequence

For transfection, cells were seeded into 6-cm cell culture dishes. At about 75% confluence, cells were transfected with constructs using Lipofectamine reagent according to the manufacturer’s instructions (Invitrogen). To construct the Flag-tagged p17 and the N-terminally and C-terminally truncated Flag-tagged p17 coding sequence of ARV S1133 strain, both p17 and p17 deleted genes were amplified from the genomic RNA of ARV S1133 strain by reverse transcription and polymerase chain reaction (RT-PCR). All primer sequences used to amplify the full-length p17 gene and p17 deleted genes are shown in Table 1. Purified PCR products were then subcloned into pcDNA3.1-flag (Invitrogen). These plasmids were cut with corresponding restriction enzymes *EcoRI* and *XhoI* and introduced into the *EcoRI* and *XhoI* sites of the pcDNA3.1-flag vector (Invitrogen). The correctness of these constructs was assessed by plasmid sequencing and by Western blot analysis by using an anti-Flag antibody.

**Table 1:** Primers used in this study for amplification of the respective targeted genes.

Gene	Accession number	Sequence (5′-3′)*	Location	Expected size (bp)
**Primers for cloning**				
p17 (Flag-tagged, full length)	AF330703	F: CGGAATTCATGCAATGGCTCCGCCATACGA (EcoR I)R: GCTCTAGATCATAGATCGGCGTCAAATCGC (Xba I)	293–314 733–712	441
p17 (His-tagged, full length)	AF330703	F: CCCAAGCTTATGCAATGGCTCCGCCATACGA (Hind III)R: CCGCTCGAGTCATAGATCGGCGTCAAATCGC (Xho I)	293–314 733–712	441
p17 (27–146)	AF330703	F: CGGAATTCACAATGGCATCATTTACTGCTATAAC (EcoR I) R: AACTCGAGTCATAGATCGGCGTCAAATCGCC (Xho I)	371–390 733–711	363
p17 (1–118)	AF330703	F: CGGAATTCACAATGCAATGGCTCCGCCATACG (EcoR I)R: AAACTCGAGTCAGGATTGAGACCCGCCATCCCAATG (Xho I)	293–313 646–623	354
p17 (119–146)	AF330703	F: CGGAATTCACAATGATCGCAGCGAAGAGAGGTCGTC (EcoR I)R: AACTCGAGTCATAGATCGGCGTCAAATCGCC (Xho I)	647–668 733–711	87
**Primers for semi-quantitative RT-PCR**				
p17 (Full length)	AF330703	F: ATGCAATGGCTCCGCCATACGAR: TCATAGATCGGCGTCAAATCGC	293–314 733–712	441
Tpr	NM_003292	F: CACTCTGATCTTGGCCAGCTTGR: CCACGGACACCTTGTCTCAATG	6637-66587115-7094	479
p53	NM_001047151	F: CCCTTCCCAGAAAACCTACCR: CTCCGTCATGTGCTGTGACT	384–403 606–587	223
PTEN	NM_001260965	F: CGAACTGGTGTAATGATATR: CATGAACTTGTCTTCCCGT	578–594 907–889	330
Rak	NM_002031	F: TGGTCAGTTCGGCGAAGTATGGR: GGCAGCCAGATCTCTGTGAATG	1173-11941518-1497	346
GAPDH	NM_002046	F: CACCACCATGGAGAAGGCTGGGGCTCAR: GGCAGGTTTCTCCAGACGGCAGGTCAG	480–506 933–907	454

F: forward; R: reverse

* Uderlines indicate the restriction sites designed in the indicated primers

### Expression of TrxA-His-p17 Fusion Protein

The p17 encoging gene of ARV S1133 strain was amplified by PCR using primer pairs as shown in Table 1. The amplified PCR products were cut with *HindIII* and *XhoI* and then introduced into the corresponding sites of the pET32a vector (Novagen, USA). The correctness of this construct was assessed by sequencing and by Western blot analysis with an anti-p17 antibody. The recombinant plasmid was then transformed into *E*. *coli* BL21 (DE3). The transformed *E*. *coli* were grown in Luria-Bertani (LB) broth with 100 mg/ml of ampicillin at 37°C to an optical density of 0.6 and then induced with 0.4 mM of IPTG for 5 h at 28°C. The cells were collected by centrifugation and then resuspended in lysis buffer (20 mM Tris-HCl pH 8; 300 mM NaCl; 0.2 mM PMSF; 10% glycerol; 5 mM imidazole) and sonicated. Cell suspension was centrifuged at 12, 000 xg for 20 minutes at 4°C. The supernatant was applied to a nickel affinity column. After washing beads with 150 ml washing buffer (20 mM Tris-HCl pH 8; 300 mM NaCl; 0.2 mM PMSF; 10% glycerol; 30 mM imidazole), the His-tagged p17 fusion protein was eluted from the affinity column with 10 ml elution buffer (20 mM Tris-HCl pH 8; 300 mM NaCl; 0.2 mM PMSF; 10% glycerol; 200 mM imidazole).

### Synthetic Peptide

To examine whether association between p17 and Tpr occurs directly, we performed *in vitro* binding assays using a synthetic peptide and purified TrxA-His-p17 fusion protein. The synthetic peptide His_6_-IAAKRGRQLD (His-p17-NLS) was synthesized by Bio Basic Inc (Markham, Canada).

### Isolation of the Fractions of the Cytosol, Nucleus, and Plasma Membrane

To investigate the distribution of p17, Tpr, p53, PTEN, p21, AKT, ERK, and cyclin D1 in the nucleus, cytoplasm, and plasma membrane in ARV-infected or p17-transfected DF-1 and Vero cells, the fractions of the cytosol and nucleus were isolated by using a CNM compartmental protein extraction kit according to manufacturer’s protocol (BioChain Inc., Hayward, USA). Cell membrane-associated PTEN was isolated according to manufacture protocol (BioChain). Briefly, cells were seeded into 6-cm cell culture dishes. At about 75% confluence, the cells were either transfected with pcDNA3.1-p17 or infected with ARV at an MOI of 5. All cultures were harvested at 24 hours post transfection or post infection. The cells were harvested after washing with ice-cold PBS twice and pelleted. They were then resuspended in 150 μl of ice cold buffer C and then the mixture was rotated at 4°C for 20 min. A syringe with a needle gauged between 26.5 and 30 was used. The needle tip was removed by bending the needle several times and only leaves the needle base on the syringe. Cell mixture was passed through the needle base 50–90 times to disrupt the cell membrane and to release the nuclei from the cells. After centrifugation at 15,000 g at 4°C for 20 min, the cytoplasmic proteins in the supernatant were collected. The pellet was resuspended with ice cold buffer W at 500 μl and then the mixture was rotated at 4°C for 5 min. The supernatant was centrifuged at 15,000 g for 20 min at 4°C. The pellet was resuspended with ice cold ice cold buffer N at 75μl and and then the mixture was rotated at 4°C for 20 min. After centrifugation at 15,000 g at 4°C for 20 min, the nuclear proteins in the supernatant were collected.

### Semi-Quantitative RT-PCR

To investigate whether ARV infection and pcDNA3.1- p17 transfection influence Tpr, p53, PTEN, and Rak transcripts, Vero cells were either transfected with pcDNA3.1-p17 or infected with ARV at an MOI of 10. All cultures were collected and lysed at 24 hours postinfection (hpi). Total RNA was isolated from the transfected or virus-infected cells using Trizol and Rneasy Mini Kit (QIAGEN) according to the manufacturer’s protocols. Total RNAs were then subjected to semi-quantitative RT-PCR as described previously [35]. The glyceraldehyde-3-phosphate dehydrogenase (GAPDH) gene was used as an internal control for normalization. The primers used for amplification of each gene are shown in Table 1.

### EGFP-Tpr Construct, shRNAs, and Akt Dominant Negative (DN) Mutant Used in this Study

To perform a rescue experiment by overexpression of EGFP-Tpr in both ARV-infected and p17-transfected vero cells, cells were transfected with either negative controls (mock and vector only) or EGFP-Tpr plasmids for 48 hours, and then either transfected with p17 or infected with ARV at an MOI of 10 for 24 hours. Cells were collected for Western blot assays. The EGFP-Tpr construct was from Addgene organization (Cambridge, MA, USA). The full-length gene of Tpr was subcloned into pEGFP-N1 vector. For immunofluorescence, Vero cells were co-transfected with both EGFP-Tpr and pcDNA3.1-p17 for 48 hours and then fixed and processed for immunofluorescence staining of p17. Colocalization of EGFP-Tpr and p17 was observed under a fluorescence microscope.

To investigate whether Akt kinase activity is directly required for p21 and affects cytoplasmic localization of p21, AKT DN mutant was used to inhibit AKT in Vero cells. The levels of p-p21 (T145) in the nucleus were analyzed. The AKT DN mutant was kindly provided by Professor Shih, National Pingtung University of Science and Technology, Taiwan. In the present study, all shRNAs and scrambled negative shRNA (Table 2) purchased from OriGene Co. (Rockville, USA) were constructed in the vector pGFP-V-RS (TR30007). Each set of shRNA kits came with four different shRNAs constructed in the pGFP-V-RS vector and was evaluated. The one given the most significant downregulation of respective protein expression was chosen and used in this study. All shRNAs used in this study are shown in Table 2. In order to confirm the role of PTEN in regulating downstream targets of AKT, ERK, mTOR, p21 and Rb, cells at 75% confluence were either transfected with PTEN gene-specific shRNA expression pGFP-V-RS vectors, scrambled shRNA or pGFP-V-RS vector. To confirm that PTEN shRNAs could effectively knockdown, the PTEN gene in DF-1 and Vero cells, the sequences of human PTEN shRNA were compared with those of chicken and monkey. To transfect cells from each well in a 6-cm cell culture dish, 2 μl of TurboFect was mixed with 1 μg of plasmid in 100 μl serum-free MEM for 20 minutes at room temperature. After 20 minutes incubation, the TurboFect/plasmid mixture was added to each well. To investigate if Rak and Rock-1 were involved in stabilizing and translocating PTEN, DF-1 and Vero cells at 75% confluence were transfected with Rak and Rock-1 shRNAs, respectively. The procedures of Rak and Rock-1 knockdown were similar to that of PTEN. To examine whether p53 is an upstream inducer of PTEN, we used p53 shRNA to knockdown p53 for investigating the p53-mediated signaling pathways [35]. After 24 hours post-transfection, the expression levels of p53 and downstream signaling molecules were analyzed by Western blot assay. PTEN stability is regulated by interaction with other proteins and it is subject to posttranslational modification, particularly phosphorylation. In addition, Tpr, LC3, and CDK4 shRNAs (Table 2) resulted in the most significant downregulation of Tpr, LC3, and CDK4 expression in Vero cells, and thus were used in virus titration assays.

**Table 2:** shRNAs used in this study.

Target gene	Cat. No.	Tube ID	Sequence (5′-3′)	Cell lines
Tpr	TG308677	GI334702 GI334704	CTCAAGATTCCATTGGAGAAGGAGTTACC GGTGAAGATAGTAATGAAGGAACTGGTAG	Vero DF-1
p53	TG320558	GI379451 GI379448	CTCAGACTGACATTCTCCACTTCTTGTTC CAGCCAAGTCTGTGACTTGCACGTACTCC	Vero DF-1
PTEN	TG320498	GI379211	GCAGTTCAACTTCTGTAACACCAGATGTT	Vero/DF-1
Rak	TG517143	GI594478	TGGTCTCAAGAGGCAGACAAGTCAGTAGT	Vero
Rock-1	TG309775	GI339094	CCAGAGTCAAGAATTGAAGGTTGGCTTTC	Vero
LC3	TG503902	GI515131	TGGACAAGACCAAGTTCCTGGTGCCTGAC	Vero
CDK4	TG320293	GI378395	CATGTGGAGTGTTGGCTGTATCTTTGCAG	Vero

### Co-Immunoprecipitation Assay

Experiments were started in serum-free medium for 2 hours and then switched to refreshing medium containing 5% FBS overnight until cell confluence reached about 75%. To explore the regulation of PTEN by Rak, β-arrestin, and Rock-1, Vero cells were transfected with respective plasmids (pcDNA3.1-p17 and pcDNA3.1) for 24 hours at 37°C. In our co-transfection assays, Vero cells were co-transfected with pcDNA3.1-p17 and the respective shRNAs for 24 hours at 37°C. The cells in 6-cm cell culture dishes were harvested and washed twice in phosphate-buffered saline (PBS) and scraped in 200 μl of lysis buffer. In order to obtain a large amount of Tpr protein in anti-ARV p17 or anti-p17 mutuants-coimmunoprecipatates for mass spectrometry analysis and Western blot assays, Vero cells in fifteen 6-cm cell culture dishes were collected. The cells were collected for immunoprecipitation by using the Catch and Release kit (Upstate Biotechnology) according to the manufacturer's protocol. Briefly, 500 μg of cellular proteins obtained as above was incubated with 4 μg of respective antibodies at 4°C overnight. The immunoprecipitated proteins were separated by SDS-PAGE, followed by Western blot, and then proteins were detected using the relevant antibodies.

### Electrophoresis and Western Blot

Western blot analysis was conducted as described previously [39]. Briefly, cells in 6-cm cell culture dishes were washed twice with PBS and then lysed with 2.5X Laemmli loading dye. Cells were harvested by scraping. Equal amounts of proteins were loaded in each lane. All protein samples were separated on either 8% SDS-PAGE gel for Tpr or 10% for other proteins, and then transferred to the PVDF membrane. For separating p17-truncated proteins in the low mass range, Tricine-SDS-PAGE was used as described previously [40]. The targeted proteins were detected using relevant antibodies, followed by a secondary antibody conjugated with horseradish peroxidase (HRP). After incubation with enhanced chemiluminescence (ECL plus) (Amersham Biosciences, Little Chalfont, England), the membrane was exposed to X-ray films (Kodak, Rochester, USA). The intensity of each protein was determined using the program Photocapt (Vilber Lourmat, France) and normalized to β-actin.

### Determination of Virus Titer

To determine the effects of Tpr, P53, PTEN, LC3, and CDK4 on ARV replication, shRNAs were used to knockdown the above targets in ARV-infected Vero cells, respectively. With the exception of CDK4, cells were transfected with the various shRNA for 6 hours, followed by ARV infection at a MOI of 5 for 24 hours. In the case of CDK 4, vero cells were infected with ARV at a MOI of 5 for 3 hours, followed by CDK 4 shRNA transfection for 18 hours. In this study, the effects of PI3K inhibitors (wortmanmin; 1 uM and LY294002; 10 uM), mTORC1 inhibitor (rapamycin; 5 uM), and inhibitor of autophagy (3MA) on ARV replication were also examined. Vero cells were pretreated with the respective inhibitor for 1 hour, followed by ARV infection at a MOI of 5 for 24 hours. Virus titer was determined as described previously [35]. Briefly, ARV-infected cell supernatant was collected. Virus titer was determined by an agar overlay plaque assay carried out in triplicate. Cells in 6-cm cell culture dishes were incubated for 1 hour with diluted virus in 100 μl serum-free MEM. The cells were then washed twice with MEM to remove unabsorbed viruses and overlaid with 2 ml of 1% agarose in MEM which contains 5% FBS and antibiotics. Plaques were checked after an incubation period of 2 days at 37°C by staining with neutral red for 3 hours.

### Statistical Analysis

The data obtained from this study were analyzed using the Student’s t-test and were expressed as averages of three independent experiments. P values of less than 0.05 were considered significantly.

## Results

### Cellular Proteins Tpr, Lamin A/C and Vimentin Coimmunoprecipatate with p17

**Fig 1:**
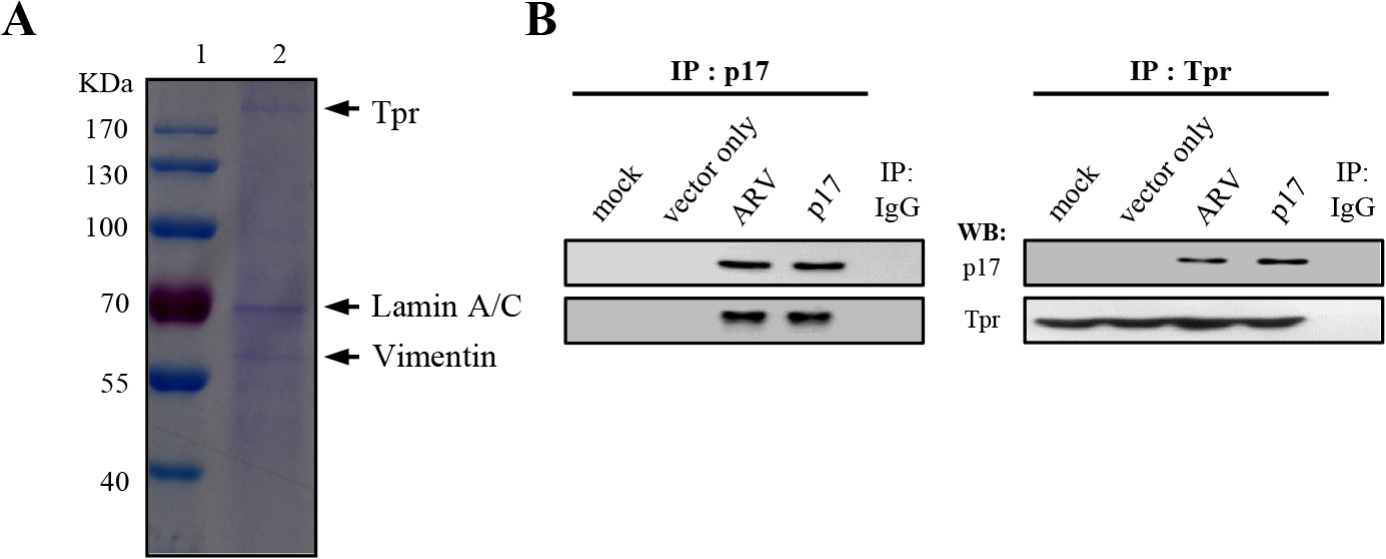
Identification of potential cellular factors that interact with p17. (A)Vero cells were transfected with pcDNA3.1-p17 plasmid for 24 hours, followed by a co-immunoprecipatation assay by p17 antibody. Cellular proteins co-immunoprecipated by p17 antibody were analyzed by SDS-PAGE. Lane 1: protein marker; lane 2: Co-immunoprecipated proteins by p17 antibody. (B) Reciprocal co-immunoprecipitation assays in both ARV-infected and p17-transfected cells were performed with either p17 or Tpr antibodies.

To identify potential cellular factors that specifically interact with p17, Vero cells were transfected with a plasmid containing p17 (pcDNA3.1-p17) for 24 hours and followed by a co-immunoprecipatation assay with the p17 antibody. To obtain large amounts of p17-coimmunoprecipatated proteins for mass spectrometry analysis, Vero cells in fifteen 6-cm cell culture dishes were harvested and concentrated. Interestingly, several cellular proteins were coimmunoprecipated by the p17 antibody, as revealed by sodium dodecyl sulphate (SDS)-polyacrylamide gel electrophoresis (Fig 1A). Three host factors (necleoporin Tpr, lamin A/C and vimentin) that coimmunoprecipated with p17 were then identified by mass spectrometry analysis. To further investigate the interplay between p17 and Tpr, Tpr with molecular mass of approximately 267 kDa that specifically coimmunoprecipated with p17 (Fig 1B) was further studied. To rule out the possibility of overexpression artifacts, reciprocal co-immunoprecipation assays were performed in both ARV-infected and p17-transfected cells as well as in mock controls. Reciprocally, p17 could also be pulled down in both ARV-infected and p17-transfected cells when an antibody against Tpr was used for immunoprecipitation (Fig 1B).

### The Nuclear Localization Signal (NLS) of p17 Is Crucial for Interaction with Tpr

**Fig 2:**
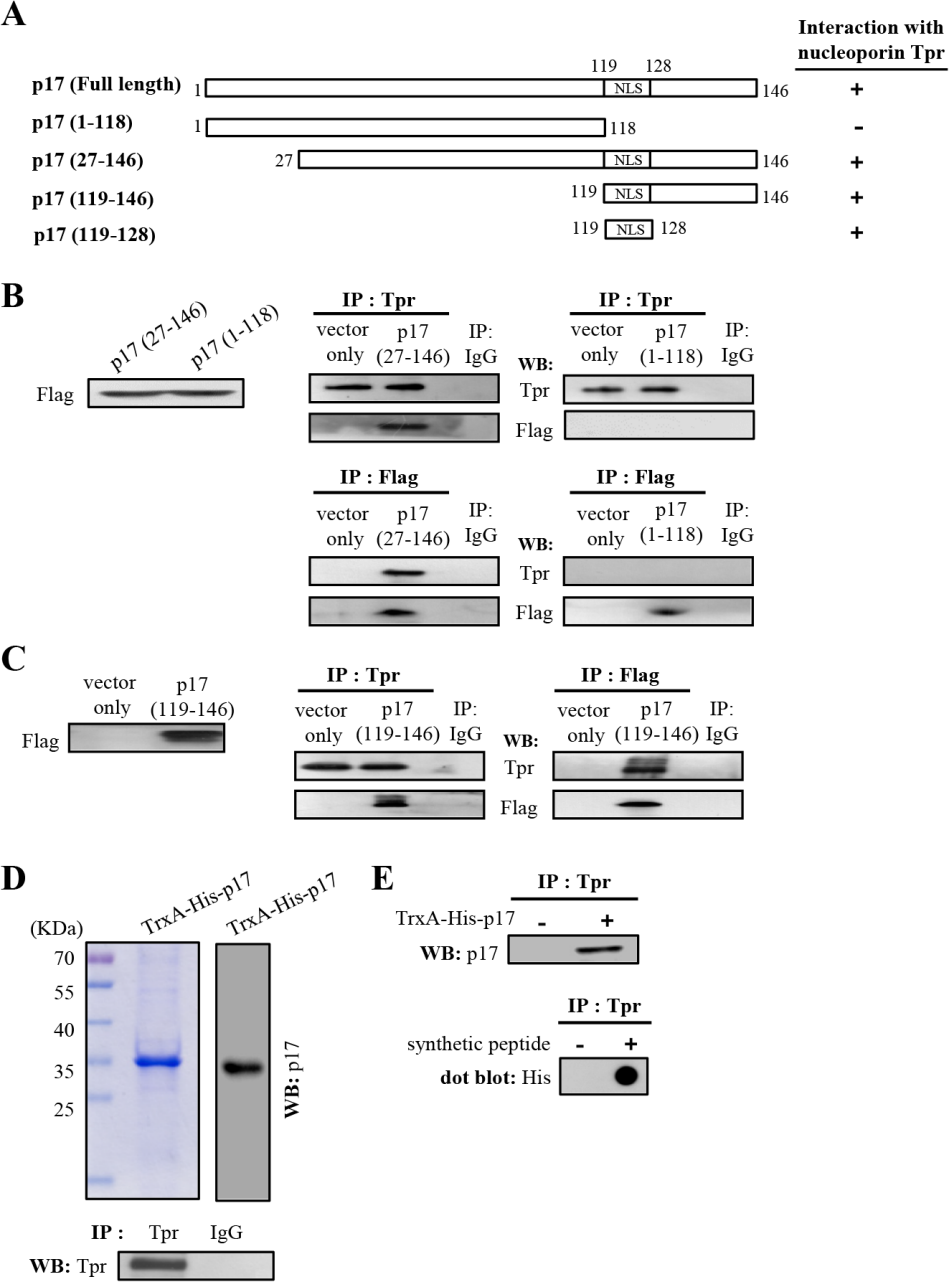
The NLS within p17 directly interacts with Tpr. (A) To map the region within p17 that was involved in Tpr binding, a series of truncated versions of Flag-tagged p17 constructs were established. Schematic representation of p17 deletion mutants is shown. The ability of the p17 and truncated p17 mutants to interact with Tpr is shown on the right hand side of the panel. +, strong binding;-, no binding. (B-C) Cellular lysates from Vero cells transfected with Flag-tagged p17 deletion vectors were immunoprecipitated with anti-Tpr and anti-Flag antibodies, respectively. Following immunoblotting analysis, Tpr and Flag-tagged p17 deletion proteins (1–118 and 27–146) (panel B) and p17 (119–146) (panel C) were detected by using Tpr and Flag antibodies. (D) Immunoprecipitation of Tpr by using an anti-Tpr antibody was performed. The immunoprecipitated Tpr proteins were separated by SDS-PAGE followed by Western blot assay using an anti-Tpr antibody (lower panel). Purified TrxA-His-p17 fusion protein was analyzed by SDS-PAGE followed by Western blot assay using an anti-p17 antibody (upper panel). Rabbit IgG was used as a negative control. (E) I*n vitro* binding assays using a synthetic peptide (His-p17-NLS, His_6_-IAAKRGRQLD) and purified TrxA-His-p17 fusion protein were performed. The synthetic peptide His-p17-NLS (lower panel) and purified TrxA-His-p17 fusion protein (upper panel) were then subjected to analysis for their binding abilities to Tpr, as revealed by Western blot and dot blot assays, as indicated. The representative data are from three independent experiments.

The above specific binding led us to further define the region within p17 for Tpr binding by using a series of Flag-tagged p17 deletion mutants. Schematic representation of p17 deletion mutants is shown in Fig 2A. The C-terminally truncated p17 mutant (1–118) and N-terminally truncated p17 mutants (27–146 and 119–146) were constructed. Cellular lysates from transfection with Flag-tagged p17 deletion vectors were immunoprecipitated with anti-Tpr and anti-Flag antibodies, respectively. Following immunoblotting assays, the Flag-tagged deleted p17 were detected (Fig 2B and 2C). The results presented in Fig 2C revealed that the deletions in p17-(27–146) and p17-(119–146), both of which still contain the NLS, did not abolish their interaction with Tpr. In contrast, the ability to interact with Tpr was abolished when the NLS within the C-terminal region of p17 (1–118) were removed [36]. To assess the consequences, we further examined whether the the C-terminal portion of p17 bearing NLS is required for interaction. Our results reveal that the NLS within p17 is critical for Tpr interaction, as revealed by reciprocal coi-mmunoprecipitation assay.

As the reciprocal co-immunoprecipitation experiments detailed above could not rule out the possibility that the interaction between p17 and Tpr occurs indirectly due to the presence of other proteins, therefore, we carried out *in vitro* binding assays by using either synthetic peptide His_6_-IAAKRGRQLD (His-p17-NLS) or purified TrxA-His-p17 fusion protein. The expressed TrxA-His-p17 fusion protein was purified by nickel column and analyzed by SDS-PAGE, followed by Western blot assay using an anti-p17 antibody (Fig 1D, upper panel). The immunoprecipitated Tpr were separated by SDS-PAGE, followed by Western blot assay using an anti-Tpr antibody (Fig 2D, lower panel). Both the synthetic peptide and purified TrxA-His-p17 fusion protein were subjected to analysis for their binding to the immunoprecipitated Tpr. Clearly, our results reveal that p17 is able to interact with Tpr, as revealed by Western blot and dot blot assays (Fig 2E, upper and lower panels).

### Both ARV Infection and p17 Transfection Suppress Tpr, Consequently Leading to p53 and p21 Nuclear Accumulation

**Fig 3:**
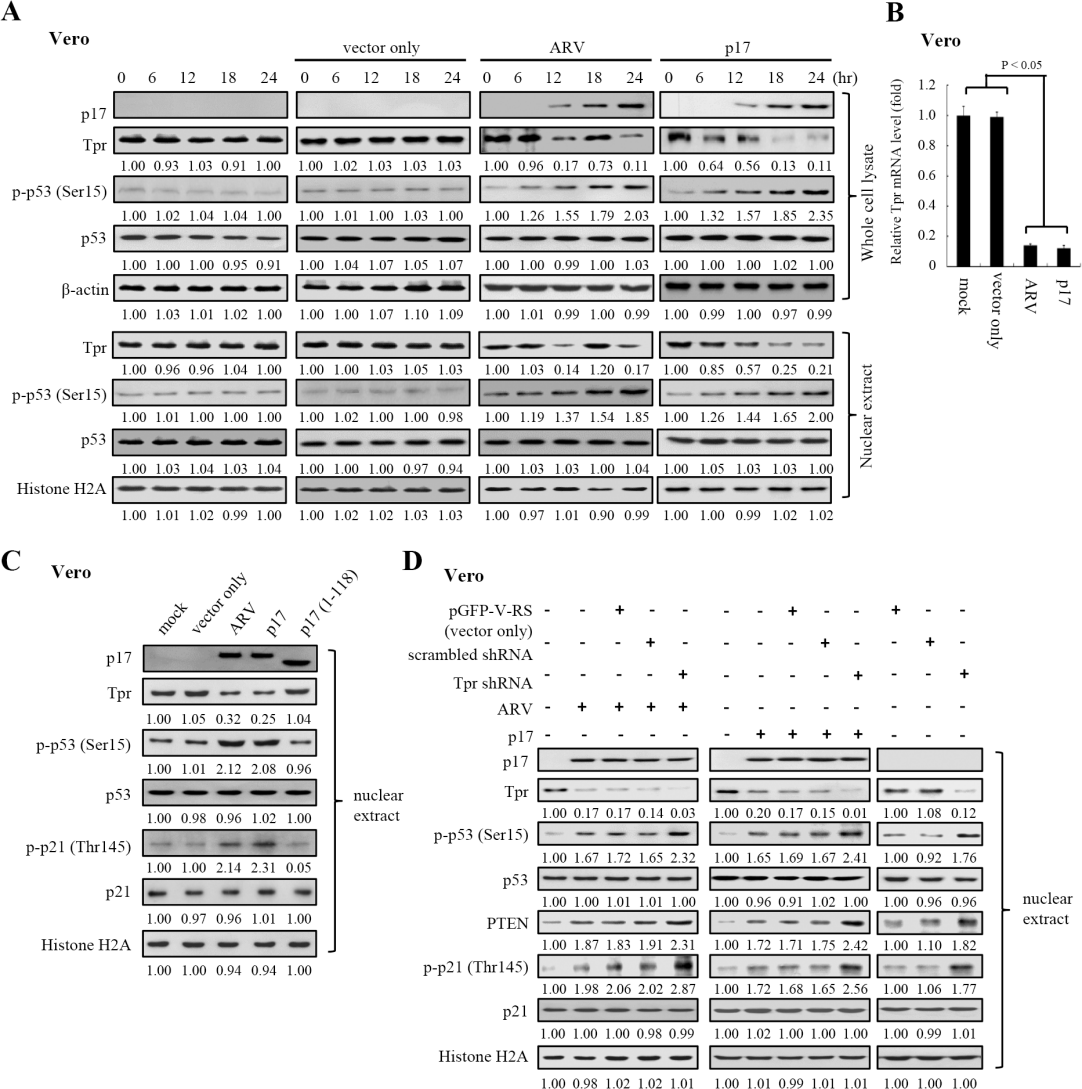
p17 functions as a Tpr suppressor leading to p53 and p21 nuclear accumulation. (A) Both Tpr and p53 levels in mock control (Vero cell only), pcDNA3.1 (mock transfection)-, pcDNA3.1-p17-transfected, and ARV-infected cells were examined. Whole cell lysates and nuclear extracts were collected at the indicated time points for Western blot assays. (B) To examine whether the Tpr transcription was down-regulated by p17, the Tpr mRNA levels in ARV-infected and pcDNA3.1-p17-transfected Vero cells were compared with those in mock treated cells. In semi-quantitative RT-PCR amplification of the p17 and Tpr genes, Vero cells were transfected with pcDNA3.1-p17 or infected with ARV at an MOI of 10. The pcCNA3.1-p17 transfected or ARV-infected cells were collected at 24 hours postinfection (hpi), and total RNAs were extracted for semi-quantitative RT-PCR. After electrophoretic separation in an agarose gel, PCR products were stained with ethidium bromide. Mock transfection (vector only) and mock infection (cell alone) were included as negative controls. Graph shown represents the mean± SD calculated from three independent experiments. (C) To study whether the p17 mutant (1–118), which does not possess a NLS, can influence the levels of p-p53 and p-p21 in the nucleus, vero cells were transfected with the p17 mutant (1–118) plasmid (negative control) for 24 hours. (D) To study whether Tpr depletion affects p53, p21, and PTEN nuclear accumulation, nuclear extracts from ARV-infected and pcDNA3.1-p17-transfected Vero cells were collected for Western blot assays. Vero cells were transfected with Tpr shRNA for 6 hours before being infected with ARV at an MOI of 10 for 18 hours. In a parallel experiment, Vero cells were co-transfected with pcDNA3.1-p17 and Tpr shRNA plasmid for 18 hours. Nuclear extracts were collected for Western blot assays using the indicated antibodies. Either actin or histone H2A was used as loading controls. The activation and inactivation folds indicated below each lane were normalized against those at 0 h (panel A) or in mock controls (cell alone) (panels C and D). The levels of indicated proteins at 0 h or in the mock controls (cell alone) were considered 1-fold.

Because of our interest in p17 modulating Tpr, we next wanted to examine whether ARV infection and p17 transfection influence the level of Tpr in both Vero and DF-1 cells. To rule out the possibility of over-expression artifacts, all assays were carried out in both ARV-infected and p17-transfected cells. Importantly, a marked decrease in Tpr was seen in both whole cell lysates and nuclear extracts in ARV-infected cells at 12 and 24 hours postinfection and in pcDNA3.1-p17- transfected cells in a time-dependent manner (Fig 3A; S1A Fig). As shown in the negative control (Fig 3B, lane 5), the levels of Tpr, p-p53, andp-p21 were not changed in p17 (1–118)-transfected cells. The above unexpected finding inspired us to further explore whether Tpr was transcriptionally regulated by p17. Thus, further analysis of the Tpr mRNA level was performed by semi-quantitative RT-PCR. Our results reveal that a corresponding decrease in the Tpr mRNA level was observed (Fig 3C; S1B Fig), suggesting that Tpr is transcriptionally downregulated by p17. The potential role of p17 in transcription of Tpr is supported by previous studies that p17 decreases cellular transcription to induce host cell shutoff [38, 39] and might activate or repress the transcription of specific genes due to its specific double-stranded DNA binding activity [36].

An increase in the level of p-p53 (Ser 15) was observed in both ARV-infected and p17-transfected cells (Fig 3A; S1A Fig). It has suggested that ATM directly phosphorylates p53 on Ser 15 and can stabilize and activate p53 through phosphorylation of both Ser 15 and Ser 20 [41, 42]. Since our team has suggested that ARV induces phosphorylation of p53 (Ser 15) by activation of ATM in response to DNA damage [43], we therefore used caffeine to block ATM and then examined the level of the p-p53 (Ser 15) in the nucleus. The level of p-p53 (Ser 15) was reversed in caffeine-treated cells (S1C Fig). Our findings are in agreement with previous observations.

**Fig 4:**
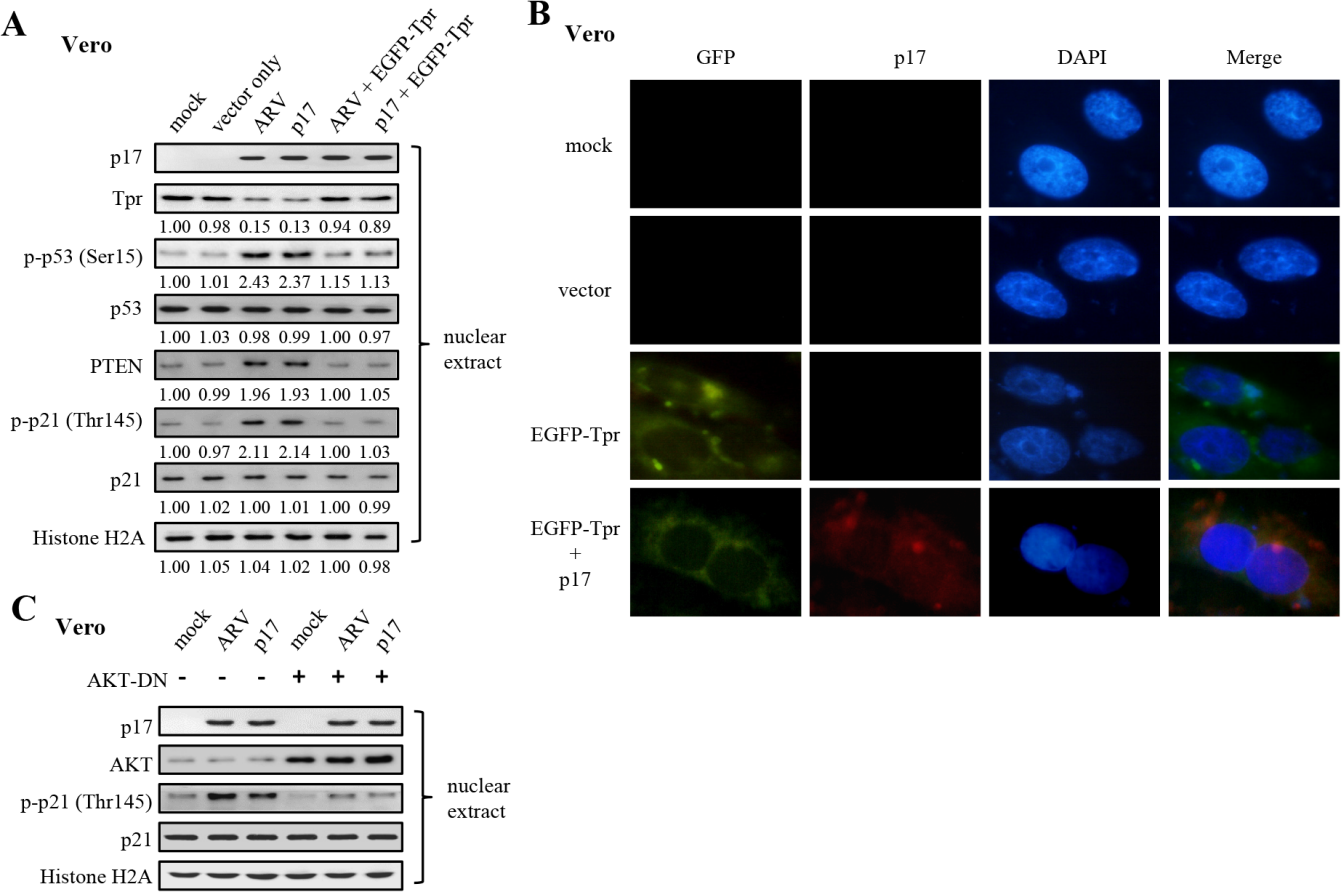
A rescue experiment by overexpression of EGFP-Tpr in ARV-infected and p17-transfected vero cells as well as examination of AKT required for p21 phosphorylation. (A) To carry out a rescue experiment by overexpression of EGFP-Tpr in ARV-infected and p17-transfected vero cells, cells were transfected with the EGFP-Tpr plasmids for 48 hours, and then either infected with ARV at an MOI of 10 or p17 transfection for 24 hours. Whole cell lysates were collected for Western blot assays. Histone H2A was used as a loading control. The activation and inactivation folds indicated below each lane were normalized against those in mock controls (cell alone). The levels of indicated proteins in the mock control were considered 1-fold. (B) Representative images of vero cells transfected with plasmids overexpressing EGFP-Tpr. Vero cells were transfected with negative controls (mock and vector only) or EGFP-Tpr plasmid for 48 hours and then visualized by immunofluorescence following nuclear DAPI staining. In addition, another set of cells were transfected with EGFP-Tpr for 48 hours and then transfected with p17 for 24 hours. Cells were fixed and processed for immunofluorescence staining of p17. Colocalization of EGFP-Tpr and p17 was observed under a fluorescence microscope. Scale bar: 10 *um*. (C) To examine whether AKT kinase activity is directly required for p21, we used an Akt DN to inhibit AKT in Vero cells. The levels of p-p21 (T145) in the nucleus were examined in ARV-infected and p17-transfected cells in the presence or absence of an Akt DN. Histone H2A was used as a loading control.

Importantly, the elevation in p-53 (S15), p-p21 (T145), and PTEN levels in the nucleus were also seen not only in Tpr-depleted cells but also in ARV-infected and p17-transfected cells (Fig 3D; S1D Fig). Furthermore, the effects of both p17-transfection and ARV-infection could be abolished by overexpression of EGFP-Tpr (Fig 4A), suggesting that p17 causes the increase in p-53 (S15), p-p21 (T145), and PTEN levels by a downregulation of Tpr. In the present study, representative images of Vero cells transfected with plasmids overexpressing EGFP-Tpr was shown in Fig 4B. Colocalization of EGFP-Tpr and p17 was observed under a fluorescence microscope (Fig 4B). Since both p21 and PTEN are the downstream targets of p53 [44, 45], upregulation of PTEN and p21 in Tpr-depleted cells was due to activation of p53. Our observations were consistent with a previous report suggesting that depletion of Tpr reduced nuclear pore formation and nucleo-cytoplasmic trafficking activities, thereby causing p53 nuclear accumulation, which induced specific downstream target genes of the p53 pathway [9, 46].

It is well-known that phosphorylation by AKT of p21 at T145 results in cytoplasmic localization leading to inactivation of p21 [47]. To further investigate whether AKT kinase activity is directly required for p21 phosphorylation and to examine whether p17 blocks p-p21 (T145) cytoplasmic localization, we used an AKT DN mutant to inhibit AKT in Vero cells. In the presence of AKT DN mutant, the levels of p-p21 (T145) in the nucleus were diminished in ARV-infected and pcDNA3.1-p17 transfected cells as well as in mock controls (cell alone) (Fig 4C, lanes 4–6), suggesting that AKT is an upstream kinase of p21. In addition, elevated levels of p-p21 (T145) in the nucleus were seen in both ARV-infected and pcDNA3.1-p17 transfected cells (Fig 4C, lanes 2–3) relative to the level in cell alone (Fig 4C, lane 1) and with AKT DN mutant treatments (Fig 4C, lanes 4–6). Our observation revealed that p17-mediated downregulation of Tpr led to the p-p21 (T145) nuclear accumulation consistent with a previous study to suggest that depletion of Tpr caused p53 and p21 nuclear accumulation [9]. Together, we conclude that p17 functions as a Tpr suppressor that causes p53 and p21 nuclear accumulation, which thereby activates p53, p21, and PTEN.

### p17 Specifically Interacts with Tpr and Impedes p53 Binding to Tpr

**Fig 5:**
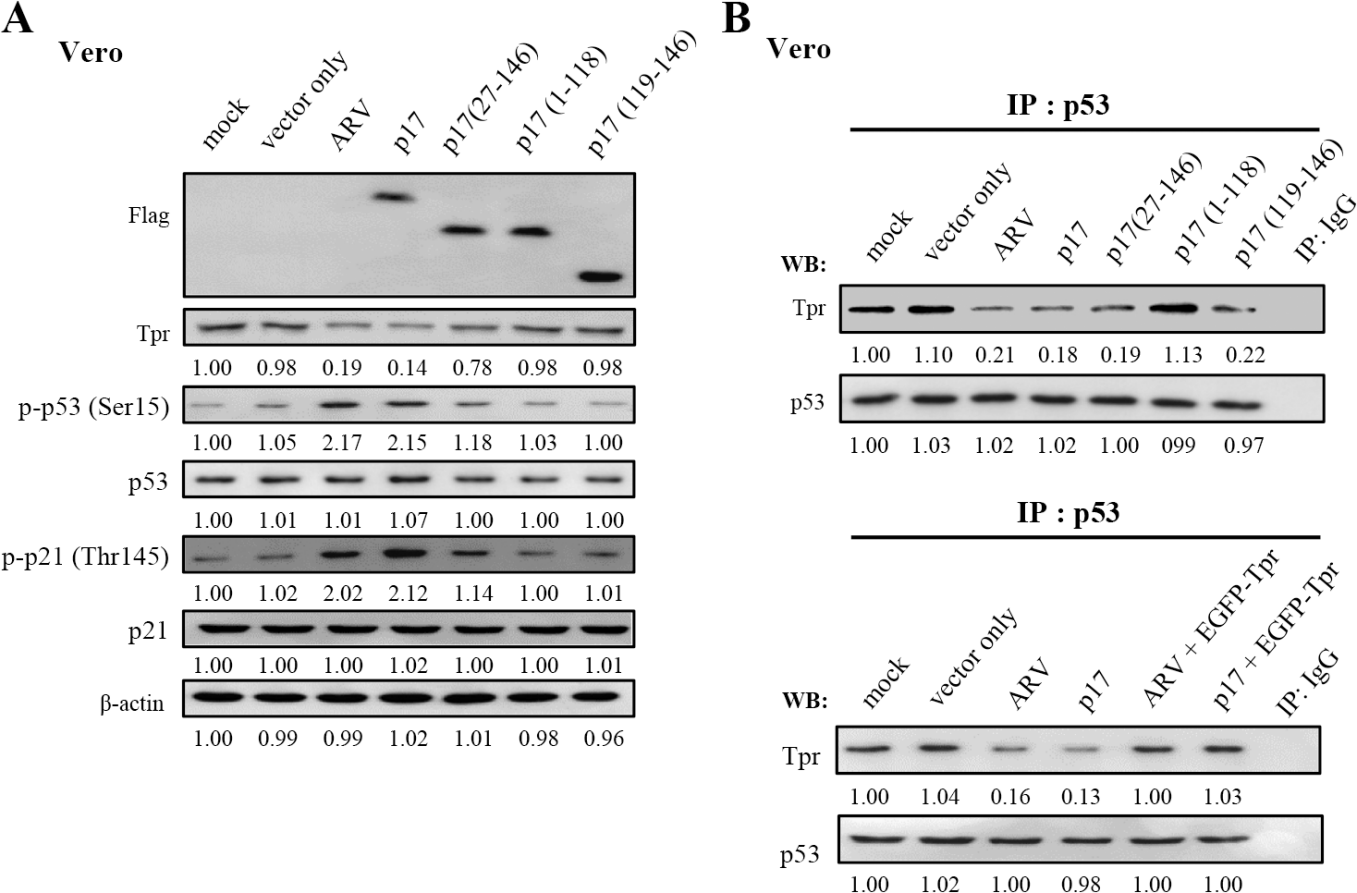
p17 specifically interacts with Tpr and blocks p53 binding to Tpr. (A) To study whether p17 and p17 mutants can downregulate Tpr expression, the levels of Tpr, p-p53, p-p21 were examined in mock-infected and ARV-infected cells as well as in pcDNA3.1-p17-, pcDNA3.1- (vector only), and p17 mutants- transfected cells. Tricine-SDS-PAGE for separating proteins in the low mass range was used. After transfection of the p17 gene and the truncated version of p17 into vero cells for 24 hours, all these proteins were separated by Tricine-SDS-PAGE, followed by Western blot assay with the anti-Flag antibody. (B) Upper panel: to examine whether p17 and p17 mutants affect the binding of p53 to Tpr, the amount of p53 and Tpr association in mock (Vero cells only), ARV-infection, pcDNA3.1-p17- and p17 mutants-transfection were examined. Lower panel: To carry out a rescue assay, cells were transfected with the indicated plasmids or infected with ARV for 24 hours. About 500 ug of cellular proteins was incubated with 4 ug of anti-p53 antibody at 4°C overnight. The immunoprecipitated proteins were separated by SDS-PAGE followed by Western blotting, and then proteins were detected with Tpr or p53 antibody, respectively. The protein levels were normalized to those for β-actin. Results were obtained from three independent experiments. The activation and inactivation folds indicated below each lane were normalized against those at mock controls. The levels of indicated proteins at the mock control were considered 1-fold.

Many studies have suggested that Tpr plays a role in nuclear protein export [3–5] and is one of the nucleoporin proteins regulating p53 nuclear-cytoplasmic trafficking [9]. Consistent with recent reports, p17-mediated downregulation of Tpr affects p53 nucleocytoplasmic trafficking, leading to p53 nuclear accumulation and activation. In this work, Tricine-SDS-PAGE for separating proteins in the low mass range was used [40]. After transfection of the Flag-tagged p17 gene and all truncated versions of p17 into vero cells for 24 hours, all these proteins were separated by Tricine-SDS-PAGE, followed by Western blot assay with the anti-Flag antibody (Fig 5A, lanes 4–7). Predictably, undetectable proteins were observed in vector only, mock-infected, and ARV-infected cells. The expression of p17 in ARV-infected cells was detected when the anti-p17 antibody was used (data not shown). As shown in Fig 5A (lanes 5–7), all truncated versions of p17 (1–118, 27–146, and 119–146) only slightly reduced the expression level of Tpr compared to negative controls and did not cause accumulation of p-p53 and p- p21 in the nucleus compared to both ARV infection and p17 transfection. This finding suggests that p17 leads to nuclear accumulation of p53 and p21 by a mechanism of transcriptional downregulation of Tpr. As the reciprocal co-immunoprecipitation experiments detailed above could not rule out the possibility that the reduced amounts of Tpr-p17 binding was due to the decrease in Tpr level, therefore, we examined the amount of binding of p53 to Tpr in cells with different treatments. In p53 co-immunoprecipitated complexes, the amount of p53-Tpr binding was dramatically reduced in ARV-infected, pcDNA3.1-p17-, and p17 mutants-transfected Vero cells relative to mock controls (cell alone and vector only) (Fig 5B, upper panel, lanes 3–4). Interestingly, the truncated versions of p17 (27–146 and 119–146) significantly diminished the amount of p53-Tpr binding (Fig 5B, upper panel, lanes 5, 7) while the amount of Tpr-p53 binding was not altered in p17 (1–118) transfection which lacks the NLS in our co-immunoprecipitation assays. Since p17 mutants (27–146 and 119–146) did not reduce the expression level of Tpr, therefore, these p17 mutants block p53 binding to Tpr by competing with p53. To further validate our findings, we employed a rescue assay by overexpressing EGFP-Tpr in both ARV-infected and p17-transfected vero cells. The effects of both ARV-infection and p17-transfection could be abolished by overexpression of EGFP-Tpr (Fig 5B, lower panel, lanes 5–6), suggesting that p17 downregulates Tpr expression level reducing the amounts of p53-Tpr binding. Taken together, we conclude that p17 functions as a Tpr suppressor by at least two independent mechanisms, which include p17-Tpr interaction and transcriptional downregulation of Tpr, thereby blocking p53 nuclear export and causing p53 nuclear accumulation.

### p17 Positively Regulates Rak and PTEN

**Fig 6:**
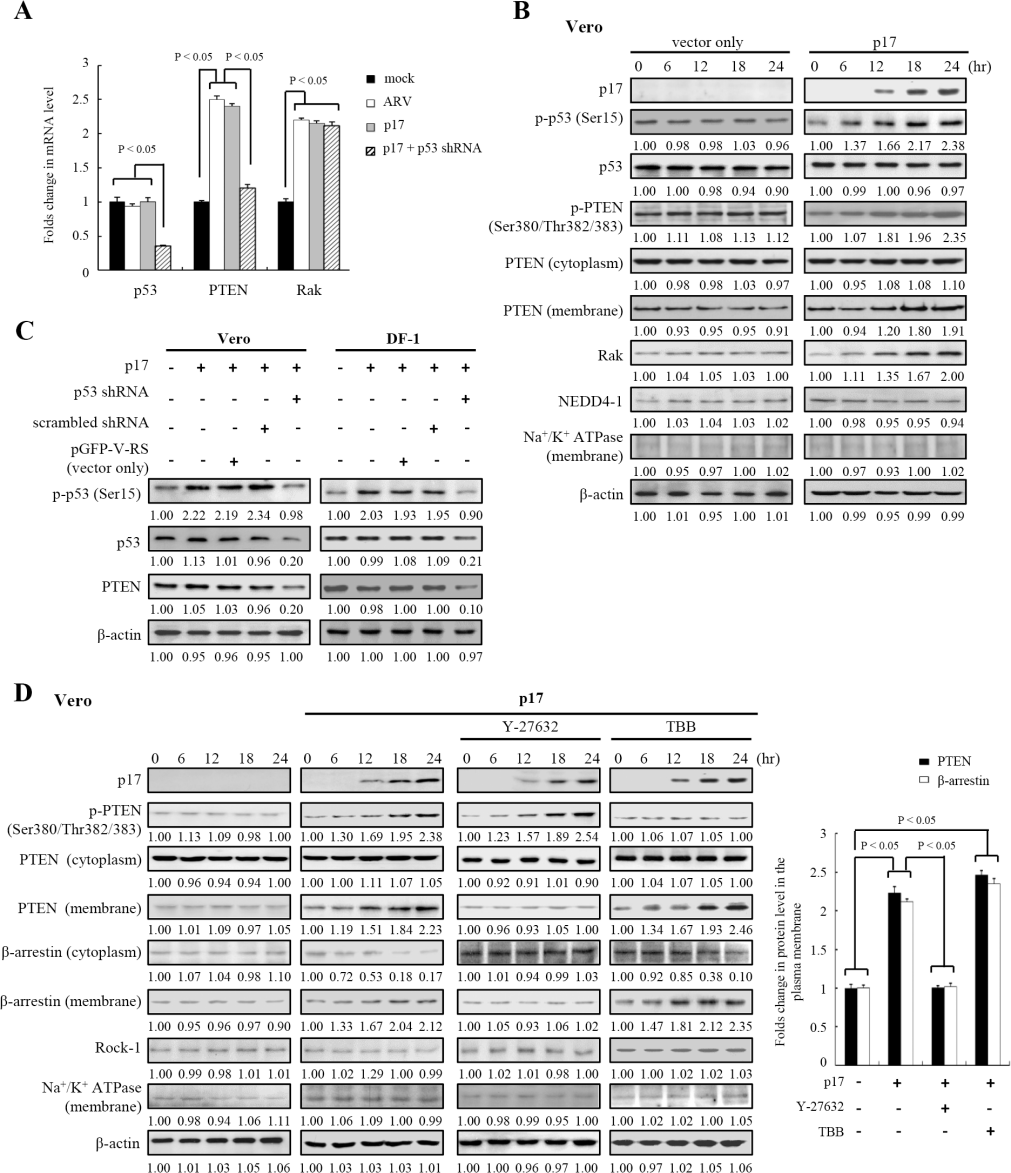
p17 positively regulates PTEN and Rak expression and drives PTEN translocation from cytoplasm into plasma membrane. (A) The p17, p53, PTEN, Rak, and GADPH mRNA levels were examined by semi-quantitative RT-PCR in ARV-infected and p17 transfected Vero cells in the presence or absence of indicated p53 shRNA. In semi-quantitative RT-PCR amplification of the p17, p53, PTEN, Rak, and GADPH genes, Vero cells were transfected with p17 or infected with ARV at an MOI of 10. The p17-transfected or ARV-infected cells were collected at either 24 hpi or 24 hours post-transfection, and total RNAs were extracted for semi-quantitative RT-PCR. After electrophoretic separation in an agarose gel, PCR products were stained with ethidium bromide. Mock control (cell alone) was included as a negative control. Graph shown represents the mean± SD calculated from three independent experiments. (B) The p17, p53, p-PTEN, cytoplasmic PTEN, membrane-associated PTEN, Rak, and NEDD4-1 levels were examined in p17-transfected Vero cells and mock control cells. (C) To confirm p53 regulated PTEN in Vero and DF-1 cells, the p-p53, p53, and PTEN levels were examined in pcDNA3.1-p17 and p53 shRNA-cotransfected cells. Vero and DF-1 cells were co-transfected with pcDNA3.1-p17, p53 shRNA, scramble shRNA, and pGFP-V-RS (vector only) for 24 hours (D) Effects of Y-27632 and TBB inhibitors on p17-mediated PTEN and β-arrestin translocation. In the presence and absence of indicated Y-27632 and TBB, the expression levels of p17, p-PTEN, cytoplasmic PTEN, membrane-associated PTEN, cytoplasmic β-arrestin, membrane-associated β-arrestin, and Rock-1 were examined in p17-transfected Vero cells and negative control (cell alone). Vero cells were pretreated with either Y-27632 (10 μM) or TBB (5 μM) for 1 hour followed by transfection with pcDNA3.1-p1 for 24 hours at 37°C. Graph on right panel shows the relative level of PTEN and β-arrestin in membrane in p17-transfected cells in the presence of Y-27632 or TBB versus cell alone. Results were obtained from three independent experiment, error bars indicate the means± SD. The protein levels were normalized to those for β-actin or Na+/K+ ATPase. The activation and inactivation folds indicated below each lane were normalized against those at 0 h (panels B and D) or mock control (panel C). The levels of indicated proteins at 0 h or in mock transfection (vector only) were considered 1-fold. The representative data are from three independent experiments.

Having demonstrated that p17 leads to p53 accumulation and activation in the nucleus, we next intended to examine if the mRNA level of p53 and its downstream molecule PTEN were upregulated by p17. In the present study, PTEN transcripts were significantly elevated in ARV-infected and p17-transfected Vero cells (up to 2.45± 0.08 and 2.4 ± 0.1 folds, respectively) (Fig 6A). It is clear that PTEN is one of the p53 downstream targets. Its transcription induction by p17 is very likely through activation of p53. No significant change of p53 transcripts was observed in ARV-infected and p17-transfected Vero cells, indicating that p17 did not affect p53 gene transcription (Fig 6A). Importantly, the p-p53 levels were elevated in both ARV-infected and pcDNA3.1-p17 transfected cells (Figs 3A and 6B; S1A Fig). Furthermore, we also found that Rak transcripts were elevated in both ARV infected and p17-transfected Vero cells (up to 2.3 ±0.1 and 2.25 ±0.05 folds, respectively) (Fig 6A). In this study, only PTEN transcripts were diminished in ARV-infected Vero cells in the presence of p53 shRNA (Fig 6A), indicating that PTEN, but not Rak, was upregulated by p53. p53 Knockdown reduced the PTEN levels, further confirming that p53 positively regulates the PTEN level in our studies (Fig 6C).

Previous studies have shown that PTEN is a substrate of casein kinase II (CK2) [48, 49]. In addition to the phosphorylation of PTEN by CK2, Rak was reported to enhance PTEN stability by direct binding and by phosphorylating cytoplasmic PTEN [50]. The previous results suggesting that p17 upregulated the Rak mRNA level directed us to further confirm whether the level of Rak was also upregulated by p17. It is worth to noting that a marked increase in Rak level was seen in pcDNA3.1-p17-transfected cells in a time-dependent manner (Fig 6B; S2A Fig; S1 Table.). No change of NEDD4-1 level was observed in pcDNA3.1-p17 transfected Vero cells (Fig 6B; S2A Fig). Interestingly, the phosphorylated cytoplasmic PTEN and membrane-associated PTEN levels were elevated in p17-transfected cells (Vero and DF-1) in a time-dependent manner (Fig 6B; S2A Fig; S1 Table.). Furthermore, the phosphorylation form of PTEN elevated by p17 was blunted by CK2 inhibitor TBB (Fig 6D; S2B Fig). Taken together, our results suggest that p17 positively regulates PTEN via activation of p53 and suppression of Tpr.

### p17 Drives β-Arrestin-Mediated PTEN Translocation from the Cytoplasm to the Plasma Membrane via a Rock-1 Dependent Manner

It is well-known that the small GTPase, RhoA, was one of the known proteins to increase PTEN lipid phosphatase activity via its downstream effector Rho kinase (ROCK) [51, 52]. We therefore examined if Rock-1 could modulate PTEN-β-arrestin interaction in cells expressing the p17 protein. Fig 6D and S2B Fig suggest that p17 enhanced PTEN and β-arrestin translocation from the cytoplasm to plasma membrane (up to 2.25± 0.15 and 2.2±0.05 folds, respectively). Their translocations in pcDNA3.1-p17 transfected cells were abrogated by Rock-1 inhibitor, Y-27632 (Fig 6D; S2B Fig), confirming the effect of the Rock-1 on PTEN-β-arrestin association and their translocation. As indicated in Fig 6D and S2B Fig, the CK2 inhibitor TBB blunted the phosphorylation of PTEN, and then increased the amount of β-arrestin and PTEN in the plasma membrane as compared to the mock control (up to 2.45±0.1 and 2.35±0.15 folds, respectively). The PTEN and β-arrestin levels in the cytoplasm and in the plasma membrane are summarized in S1 Table. Collectively, our results suggest that p17 facilitates β-arrestin-PTEN translocation from the cytoplasm to the plasma membrane via a Rock-1-dependent manner.

### p17 Stabilizes PTEN by Promoting Rak Binding to PTEN and by Increasing the Phosphorylation of Cytoplasmic PTEN to Protect It from NEDD4-1 Targeting

**Fig 7:**
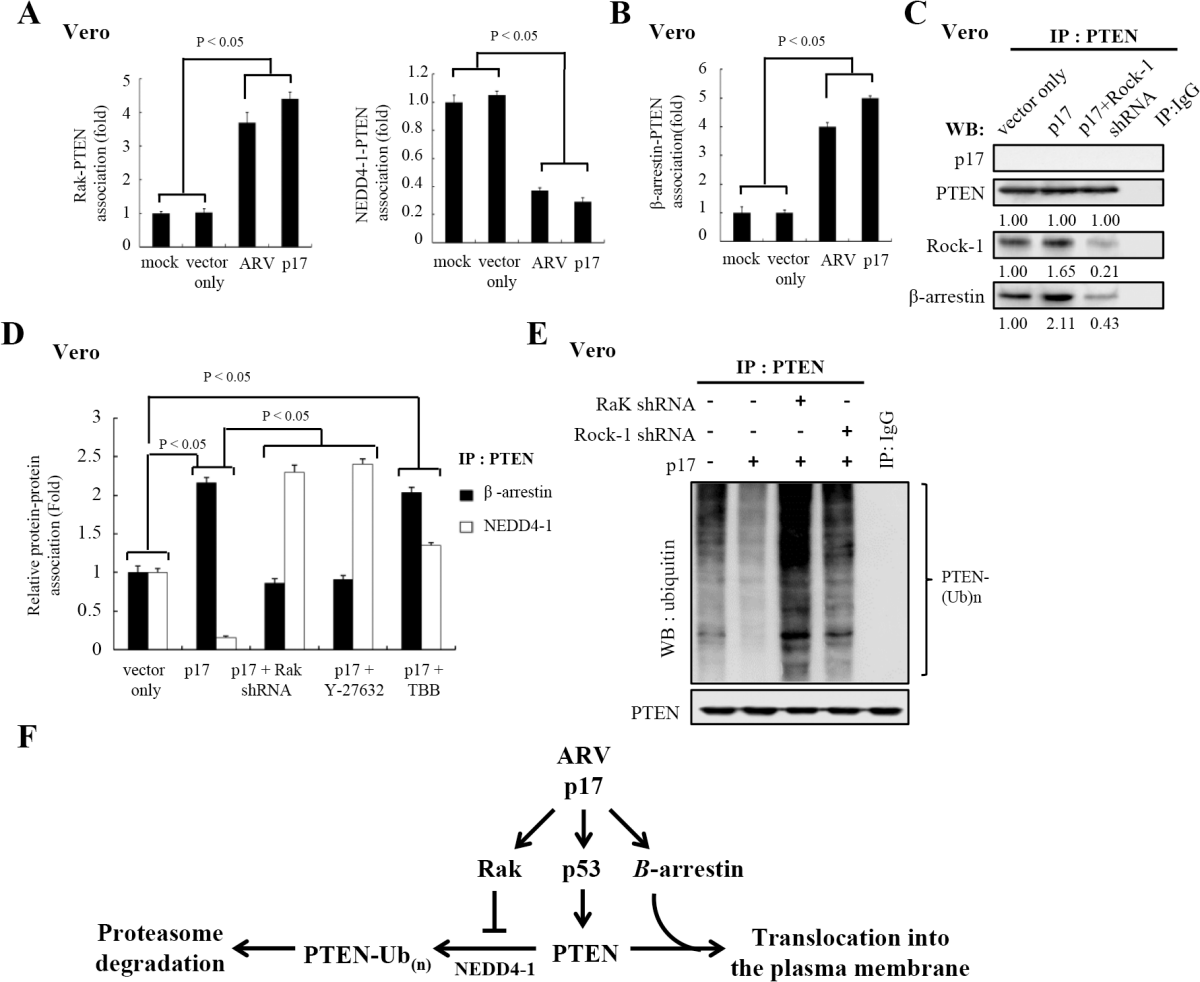
p17 stabilizes PTEN by promoting Rak-PTEN association and by stimulating phosphorylation of PTEN to protect PTEN from NEDD4-1 targeting. (A) In reciprocal co-immunoprecipation experiments, the amounts of Rak and PTEN association were examined in ARV-infected or p17-transfected cells. Western blot assay of PTEN, and NEDD4-1 contained in PTEN or Rak immunoprecipates was carried out. The p17-transfected, mock-transfected, mock-infected, and ARV-infected cells were collected at either 24 hpi or 24 hours post-transfection for Western blot assays. Data shown represent the mean± SD calculated from three independent experiments. The amounts of Rak-PTEN and NEDD4-1-PTEN associations were normalized against those at mock-transfection and mock-infection. The level of mock controls was considered 1-fold. (B) In reciprocal co-immunoprecipation experiments, the binding of β-arrestin to PTEN was examined in either ARV-infected or p17-transfected Vero cells. Western blot of β-arrestin, PTEN, and NEDD4-1 contained in PTEN or β-arrestin immunoprecipates was carried out. Data shown represent the mean± SD calculated from three independent experiments. The amounts of β-arrestin-PTEN associations were normalized against those at mock-transfection and mock-infection. The level of mock controls was considered 1-fold. (C) In coimmunoprecipation experiments, the binding of β-arrestin to PTEN was examined in p17-transfected Vero cells. Western blot of PTEN and β-arrestin contained in PTEN immunoprecipates was carried out in presence and absence of Rock-1 shRNA. Rabbit IgG was used as a negative control. The representative data are from three independent experiments. The activation and inactivation folds indicated below each lane were normalized against those at vector only. The level of indicated proteins at vector only was considered 1-fold. (D) The interaction between PTEN and β-arrestin as well as between NEDD4-1 and PTEN were examined in p17-transfected and mock-transfected Vero cells. Proteins immunoprecipated with an anti-PTEN antibody from Vero cell lysates treated with Rak shRNA, Y-27632, and TBB, respectively were resolved by SDS-PAGE and immunbloted with the indicated antibodies. Data shown represent the mean± SD calculated from three independent experiments. (E) Cells were co-transfected with pcDNA3.1-p17 plasmid with either Rak or Rock-1 shRNA for 24 hours. The cells were harvested and washed twice in PBS buffer and scraped in 200 μl of lysis buffer. About 500 ug of cellular proteins was incubated with 4 ug of anti-PTEN antibody at 4°C overnight. The immunoprecipitated proteins were separated by SDS-PAGE followed by Western blot assay, and then proteins were detected with anti-ubiquitin antibody. Rabbit IgG was used as a negative control. The representative data are from three independent experiments. (F) A model illustrates the PTEN regulation by p17.

A previous study on PTEN protein stability has demonstrated that it is regulated by ubiquitin-mediated proteasome degradation through the E3 ligase NEDD4-1 [53]. Phosphorylation of PTEN by either CK2 or Rak was reported to enhance PTEN stability [48–50]. To test whether p17 stabilizes PTEN by enhancing Rak binding to PTEN, reciprocal coimmunoprecipation assays were carried out. Our results revealed that the increase in the interaction between Rak and PTEN was seen in ARV-infected and p17-transfected cells (up to 3.8±0.15 and 4.5±0.2 folds, respectively) (Fig 7A), thereby inhibiting NEDD4-1 targeting to PTEN (down to 2.8±0.3 and 3.1± 0.25 folds, respectively) (Fig 7A). Furtherore, an increase in β-arrestin-PTEN association was also observed (up to 3.5±0.25 and 6±0.2 folds, respectively) (Fig 7B), accompanied by a decreased amount of NEDD4-1-PTEN association.

To further confirm whether p17 stabilizes PTEN by enhancing the Rak-PTEN association and by promoting the phosphorylation of cytoplasmic PTEN, both Rock-1 and Rak shRNAs, as well as Rock-1 and CK2 inhibitors were used. In the present study, Rock-1 knockdown and Y-2763 treatment decreased β-arrestin binding to PTEN (Fig 7C and 7D). Importantly, the decreased amount of β-arrestin-PTEN association in our immunprecipation assays was also seen in Rak shRNA-treated cells (Fig 7D). On the other hand, the amount of NEDD4-1 targeting to PTEN was elevated in Rak shRNA- and Y-2763-treated cells relative to vector only (up to 2.35±0.15 and 2.45±0.1 respectively (Fig 7D). In TBB-treated cells, elevation of both β-arrestin-PTEN and NEDD4-1-PTEN associations were seen (Fig 7D). It was also found that PTEN did not co-immunoprecipitate with p17 (Fig 7C and 7D). To validate our data, we next wanted to examine the relative level of ubiquitination of PTEN in p17-transfected cells. Notably, Rak depletion significantly increased the PTEN targeting by ubiquitin in our co-immunoprecipation assays (Fig 7E, lane 3). A similar trend was also observed in Rock-1 knockdown treatment, showing an increased amount of PTEN targeted by ubiquitin in the co-immunoprecipation assay (Fig 7E, lane 4). To clearly show the regulation of PTEN by p17, a model depicting the stabilization and activation of PTEN is shown in Fig 7F. In the present study, we demonstrate for the first time that p17 also acts as a positive regulator of PTEN by stabilizing PTEN by elevating Rak-PTEN binding to prevent it from E3 ligase NEDD4-1 targeting. Furthermore, p17 is able to promote arrestin-mediated PTEN translocation from the cytoplasm to the plasma membrane via a Rock-1-dependent manner.

### p17 Negatively Regulates ERK and Cyclin D1 and Positively Regulates Rb and p21 through a Tpr/p53/PTEN-Dependent Signaling Pathway

**Fig 8:**
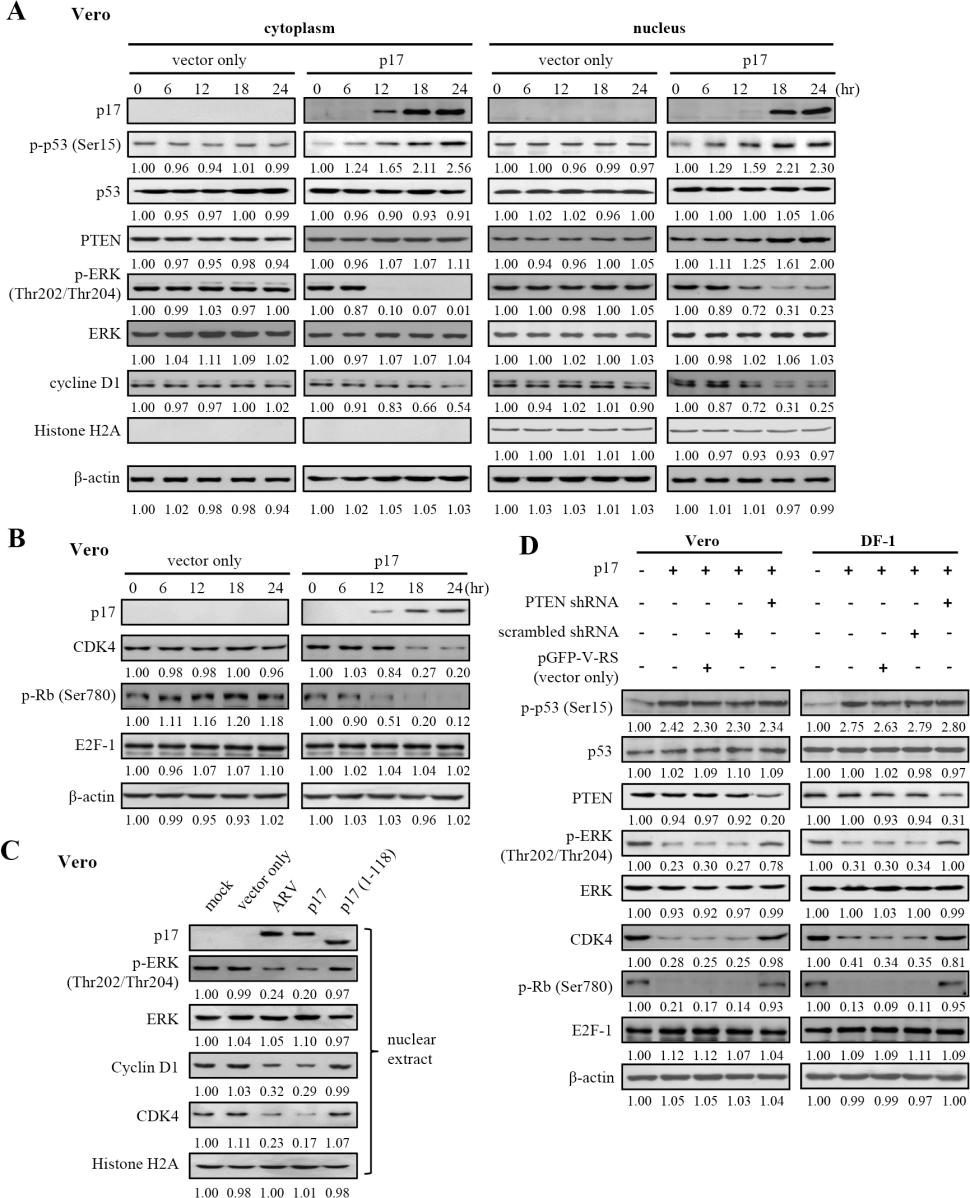
p17 negatively regulates ERK and cyclin D1 and positively regulates Rb through Tpr/p53/PTEN and Tpr/p53/p21 pathways. (A-B)Vero cells were transfected with pcDNA3.1-p17 or pcDNA3.1 (vector only) for 24 hours. The results of Western blot analysis of cellular fractions from the cytoplasm and nucleus (A) or whole cell lysates (B) at the indicated time points are shown. Phosphorylation and protein levels were determined by immunoblotting with the indicated antibodies. (C) To study whether the p17 mutant (1–118) which does not possess a NLS can affect the levels of p-ERK, cyclin D1, and CDK4 in the nucleus, vero cells were transfected with the negative control p17 mutant (1–118) for 24 hours. (D) To confirm whether PTEN is the upstream signaling that regulates the above molecules, shRNA-mediated blockade of PTEN was performed. Vero and DF-1 cells were co-transfected with pcDNA3.1-p17 and PTEN shRNAs for 24 hours followed by Western blot analysis with indicated antibodies. In the negative controls, cells were also co-transfected with p17 and respective negative controls (pGFP-V-RS and Scramble shRNA plasmids) for 24 hours. Phosphorylation and protein levels were determined by immunoblotting with the indicated antibodies. The protein levels were normalized to that for β-actin (panels A, B, C) or Histone H2A (panel A). The activation and inactivation folds indicated below each lane were normalized against those at 0 h or in mock control. The levels of indicated proteins at 0 h or in mock control (cell only) were considered 1-fold. The representative data are from three independent experiments.

As seen from the the previous data showing that p17 activated PTEN, we next wanted to investigate whether its downstream molecules were suppressed. Nuclear PTEN is essential for tumor suppression and its nuclear import is mediated by its monoubiquitination [54]. In this study, the elevation in nuclear PTEN was found in p17-expressing cells. Consistent with a previous study [55], the increased levels of PTEN were correlated with a decrease in p-ERK and cyclin D1 levels in the nucleus in a time-dependent manner, as revealed by Western blot (Fig 8A; S3A Fig). Based on current knowledge, control of the G1/S phases of cell cycle transition is largely a matter of regulating a set of specific CDK activities. In mammalian cells, the G1/S specific CDK activities are composed of complexes between D type cyclins and either CDK4 or CDK6. A previous study has suggested that cyclin D1-CDK4 phosphorylates Rb *in vivo* [56]. Kitagawa and co-workers suggested that the S780 in Rb is phosphorylated during the G1 phase in a cell cycle-dependent manner and that Rb phosphorylated at S780 cannot bind to E2F-1 *in vivo* [56]. Importantly, this study further demonstrates that p17 negatively regulates Tpr leading to positive regulation of p53, PTEN, and p21 with the concomitant decrease in p-ERK, cyclin D1, and CDK4 levels in both Vero and DF-1 cells in a time-dependent manner, thereby resulting in Rb (S780) dephosphorylation/activation (Fig 8A and 8B; S3A and S3B Figs). The level of E2F-1 was not altered. In addition, a dramatic reduction in the cyclin D1 level in the cytoplasm and the nucleus was seen in p17-transfected cells (Fig 8A; S3A Fig). The levels of p-ERK, cyclin D1, CDK 4, and p-Rb were reversed in p17 (1–118)-transfected cells (Fig 8C) as compared to p17 transfection. The levels of PTEN and its downstream molecules are summarized in S1 Table.

To further confirm the effects of Tpr and p53 on PTEN and its downstream signaling of ERK and CDK4, PTEN depletion by shRNA was performed in two independent p17-transfected cells. In Fig 8D (right and left panels, lane 5), the level of PTEN was diminished, as revealed by Western blot assays. Findings presented in this study reveal that PTEN knockdown reversed the level of p-ERK, CDK4, and p-Rb (Fig 8D, right and left panels, lane 5). Consistent with a previous study by Planchon et al. [55], depletion of PTEN reversed the levels of p-ERK and cyclin D1, indicating that PTEN downregulates ERK leading to a decrease in cyclin D1 levels. Additionally, the levels of p53 and phosphorylation form of p53 were not altered in PTEN shRNA-treated cells.

### p17 Negatively Regulates PI3K/AKT/mTOR Signaling Pathway through Suppression of Tpr and Activation of p53 and PTEN

**Fig 9:**
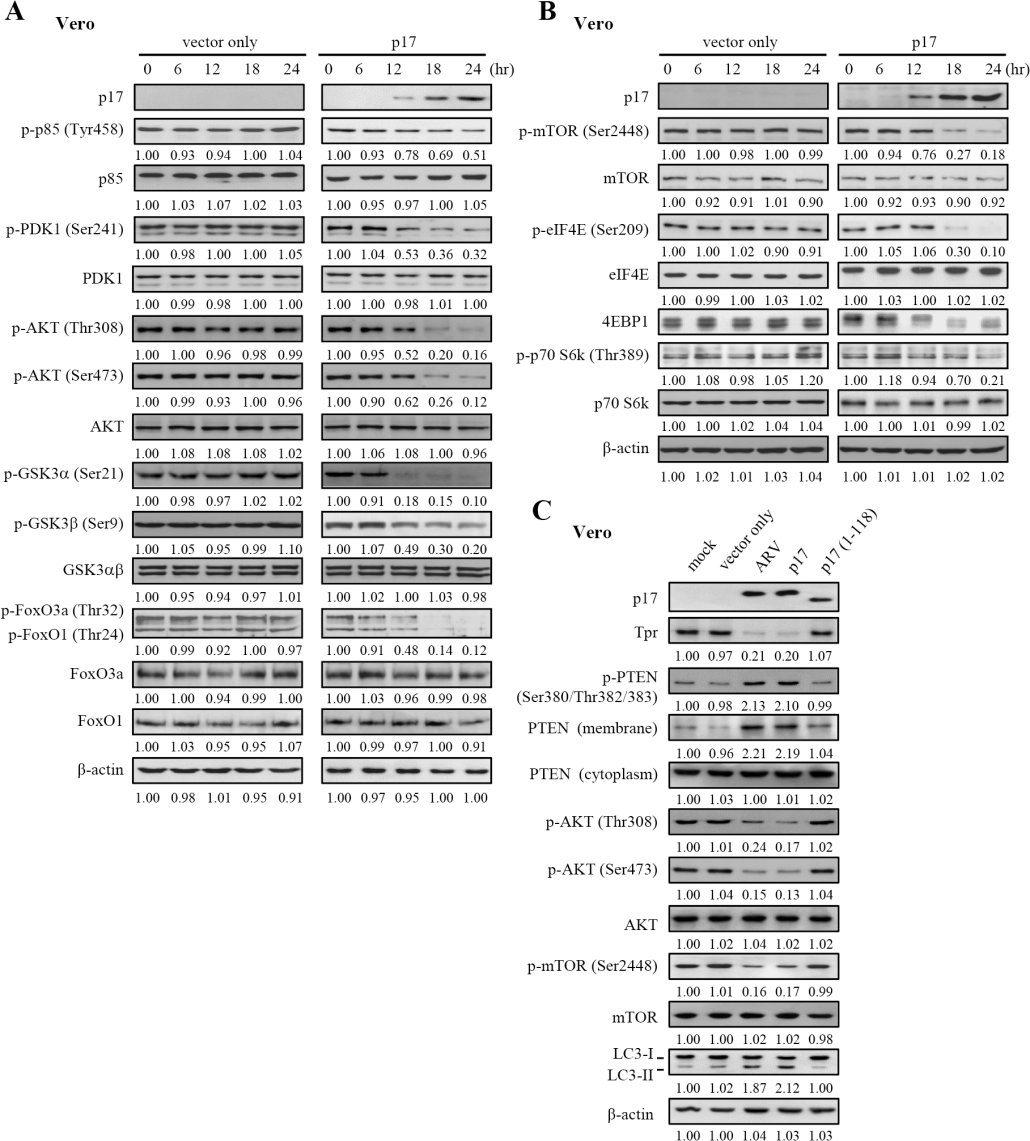
p17 negatively regulates PI3K/AKT/mTOR signaling pathway. (A-B) Vero cells were transfected with pcDNA3.1-p17 and pcDNA3.1 (vector only) plasmid, respectively for 24 hours. Whole cell lysates were collected at the indicated time points, and the levels of PI3K and its downstream molecules were examined by Western blot assay with the indicated antibodies. (B)Vero and DF-1 cells were co-transfected with both pcDNA3.1-p17 and p53 shRNAs for 24 hours, followed by Western blot analysis with indicated antibodies. Cells were also co-transfected with pCDNA3.1- p17 and respective negative controls (scrambled shRNAs and pGFP-V-RS vector) for 24 hours. (C) To study whether the negative control p17 mutant (1–118) can affect the levels of p-PTEN, p-AKT, p-mTOR, and LC3-II, vero cells were transfected with the p17 mutant (1–118) plasmid for 24 hours. Similar results were obtained from three independent experiments. The protein levels were normalized to those for β-actin.The activation and inactivation folds indicated below each lane were normalized against those at 0 h or mock. The levels of indicated proteins at 0 h or mock were considered 1-fold.

As its lipid phosphatase activity by converting PIP3 to PIP2, PTEN prevents AKT activation. Therefore, we next examined the effect of p17 on promoting β-arrestin-mediated PTEN regulation by looking at p-AKT levels. The plasma membrane-associated PTEN level (Fig 6B) was elevated with concomitant PDK-1 and AKT dephosphorylation/inactivation in total cell lysate (Fig 9A; S4 Fig) in a time-dependent manner. An earlier study has suggested that mTORC2 phosphorylates AKT at S473 in its C-terminal hydrophobic motif, which in conjunction with PDK1-regulated phosphorylation at T308, drives full activation of AKT [57]. Our results reveal that p17 dramatically reduces phosphorylation of AKT at T308 and S473 in total cell lysate (Fig 9A; S4 Fig) as compared to the negative control (vector only). It is important to note that although p21 phosphorylation at T145 is shown to be dependent on AKT (Fig 4C) and that at least eighty five percent of p-AKT levels are reduced by p17 (Fig 9A and 9C), the elevated levels of p-p21 in the nucleus in both ARV-infected and p17-transfected cells (Fig 4C, lanes 2–3) are likely due to p21 phosphorylation by the residual activated AKT and nuclear accumulation.

Decreased levels of upstream of AKT (p-PI3K, p85 T458) were also observed (Fig 9A). In response to PTEN-mediated Akt inactivation by p17, at least two downstream targets of AKT, including GSK3α/β and FoxO1/3a [58–63], were examined in this study. The phosphorylation levels of AKT downstream targets were dramatically abolished in p17-transfected cells in a time-dependent manner (Fig 9A; S4 Fig). mTORC1 plays the primary role in regulating autophagy. Inactivation of TORC1 will allow an increase in autophagy activity. As an indicator of mTOR activity, we measured the phosphorylation level of p70S6 kinase at the Thr389 site. The phosphorylation levels of p70S6 kinase were abolished by p17 protein (Fig 9B). Decreased amounts of 4E-BP1 and dephosphorylation of the cap-binding protein eIF4E were also seen in cells expressing p17 protein (Fig 9B), thereby causing host cellular translation shutoff. Similar to the negative controls, the levels of Tpr, p-PTEN, p-Akt, p-mTOR, and LC3-II were reversed in p17 (1–118) mutant-transfected cells (Fig 9C, lane 5) as compared to ARV infection or p17 transfection. Our results reveal that the NLS within C-terminal of p17 is important for activating PTEN and inhibiting mTOR as well as for inducing formation of LC3-II (autophagy marker). The levels of PI3K and AKT and their downstream molecules are summarized in S1 Table. This study suggests that p17 causes host cellular translation shutoff via inactivation of eIF4E and p70S6k through the Tpr/p53/PTEN/AKT/mTOR pathway.

To further confirm that p17-modulated inactivation of AKT/mTORC1 signaling pathway occurs through activation PTEN, the expression of PTEN was silenced in two independent pcDNA3.1-p17 transfected cells using PTEN shRNA. The decrease in PTEN, p-PDK1, p-AKT, p-GSK3α/β, p-FoxO1/3a, and p-mTOR levels were seen in cells only transfected with pcDNA3.1-p17 plasmid (S5 Fig). These effects could be reversed in cells treated with PTEN shRNA in both vero and DF-1 cells (S5 Fig left and right panels, lane 5). In this study, original images of all blots in this study with molecular weight are shown in S6–S8 Figs.

### Depletion of Tpr and CDK4 Is Beneficial for Virus Replication

**Fig 10:**
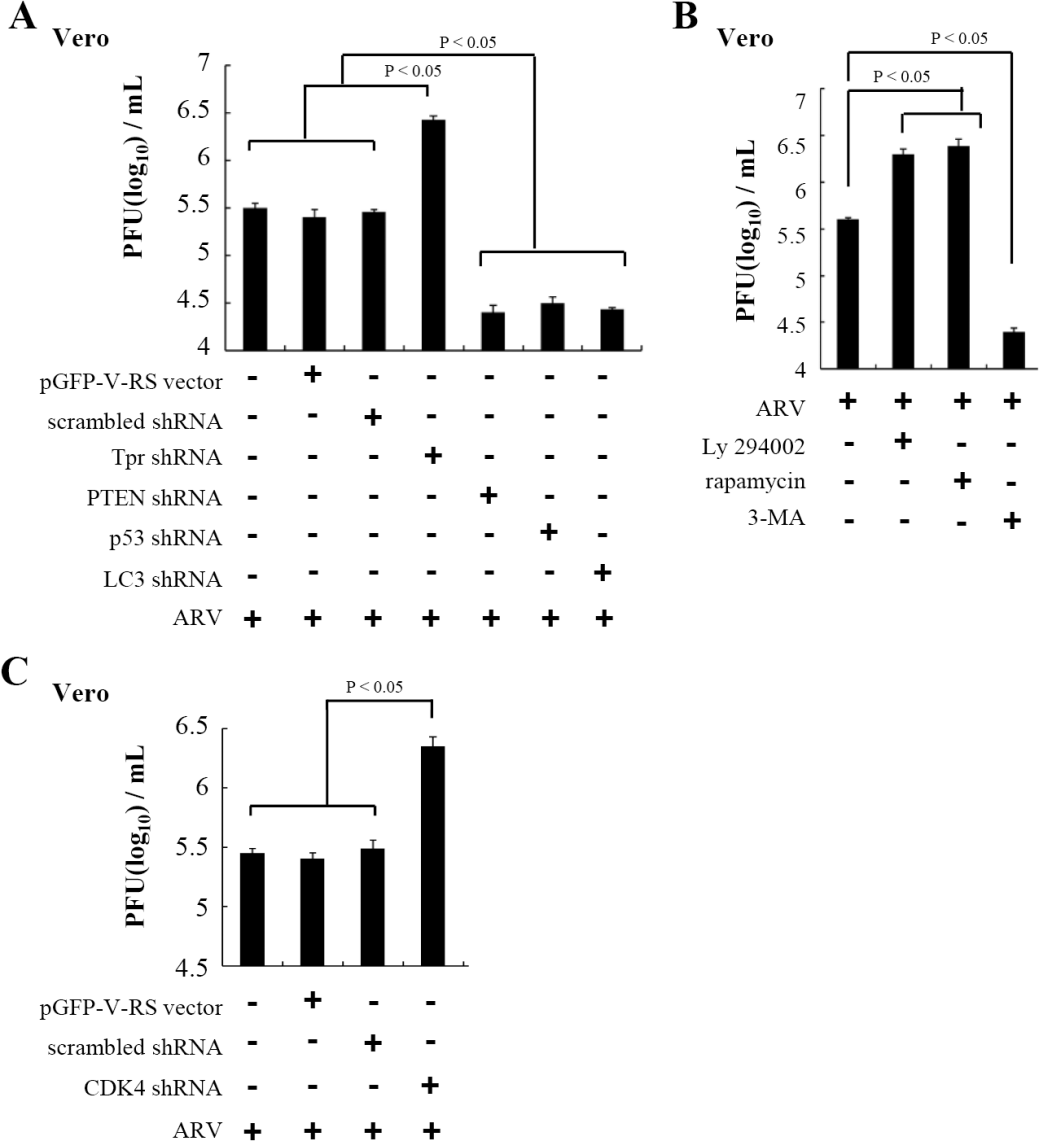
Knockdown of Tpr is beneficial for virus replication. (A)To determine the effect of Tpr on ARV replication, depletion of Tpr with shRNA was carried out in ARV-infected Vero cells. The downstream molecules (p53, PTEN and LC3) of Tpr were also depleted using the respective shRNAs. All data shown represent the mean± SD calculated from three independent experiments. (B) In the present study, the effects of PI3K inhibitor (LY294002, 10 uM), mTORC1 inhibitor (rapamycin; 5 uM), and inhibitor of autophagy (3MA) on ARV replication were examined. Data shown represent the mean± SD calculated from three independent experiments. (C) In the case of CDK 4, vero cells were infected with ARV at a MOI of 5 for 3 hours, followed by CDK 4 shRNA transfection for 18 hours. All data shown represent the mean± SD calculated from three independent experiments.

As previously demonstrated, p17 is a Tpr suppressor leading to activation of p53, p21, and PTEN. To further determine the impact of Tpr on virus replication, Tpr knockdown was performed. Importantly, Tpr depletion increased virus production (Fig 10A). Conversely, knockdown of p53, PTEN, and autophagy markers (LC3) diminished virus yield (Fig 10A). Moreover, we also investigated the effects of PI3K inhibitor (LY294002), mTORC1 inhibitor (rapamycin), and autophagy inhibitor (3MA) on ARV replication. As presented in Fig 10B, inhibition of PI3K and mTORC1 by inhibitors increased virus titers, but inhibition of autophagy by 3MA reduced virus yield. These results are in consistent with our previous study [39]. Aside from this, a decrease in CDK4 level was observed in p17-transfected cells (Fig 8B; S3B Fig), and so we therefore also investigated the effect of CDK 4 on virus replication. In this work, expression of CDK4 was silenced. After vero cells were infected with ARV for 3 hours, CDK 4 knockdown in vero cells increased virus yield (Fig 10C). Collectively, our findings in this study reveal that p17-modulated suppression of Tpr and subsequent activation of upstream signaling (p53, PTEN, and p21) induces cell cycle arrest and autophagosome formation, which in turn enhancing virus replication [37–39, 64].

## Discussion

The discovery that p17 acts as a Tpr suppressor offers important insight into its functionality in modulating the upstream signaling molecules p53, PTEN, and p21 and their downstream targets. An earlier study has suggested that Tpr depletion increases p53 and p21 nuclear accumulation and facilitates autophagy [9]. In the present study, we describe a novel function for p17 whereby it acts as a Tpr suppressor, impacting Tpr function, and thus causing p53 and p21 nuclear accumulation. Our study identifies Tpr as a new player in the regulation of the p53, PTEN, and p21 by p17-mediated Tpr suppression. The results provide conclusive evidences showing that p17 inhibits Tpr by direct interaction with and downregulation of Tpr transcription. p17 inhibition of Tpr thereby activates the upstream signals p53, PTEN, and p21 and subsequently inactivates the downstream signaling pathways PI3K/AKT/mTOR and ERK as well as cyclin D1 and CDK4. This work clarifies the p17-mediated cross-talk between Tpr and p53/PTEN/AKT/mTOR and p53/PTEN/ERK signaling pathways. In this report, detailed studies were undertaken to define the regions involved in the interaction between p17 and Tpr. By using Flag-tagged p17 deletion fusion proteins combined with a synthetic peptide, the Tpr-binding domain was defined within the C-terminal region of p17. In our studies, we have identified that amino acid residues 119IAAKRGRQLD128 of p17 are required for Tpr binding. The nuclear localization of p53 is regulated by a number of mechanisms. In this study, we suggest that p17 causes nuclear accumulation of p53 by competing with p53 binding to Tpr and by masking the nuclear export (NES) due to phosophorylation of Ser15 in p53 [65], which in turn leads to inhibition of p53 nuclear export and causes p53 nuclear accumulation, thereby activating its downstream targets p21 and PTEN [44, 45].

The effect of p17 on PTEN was also extensively studied. The canonical PTEN pathway has attracted much attention due to its fundamental contribution to human malignancies. It is clear that PTEN stability can be modulated through the C-terminal tail [66, 67] which is phosphorylated upon a cluster of serine and threonine residues such as S380, T382, T383, and S385. A recent study on the regulation of PTEN by Rak has demonstrated that Rak positively regulates PTEN protein stability by phosphorylating PTEN on Tyr 336, which in turn prevents PTEN ubiquitination and degradation [50]. An interesting finding obtained in this study was that p17 is able to upregulate Rak gene transcription and protein expression, which in turn promotes Rak binding to PTEN and blocks NEDD4-1 targeting to PTEN [53]. In our studies, we have identified the mechanisms that p17 stabilizes PTEN by stimulating phosphorylation of cytoplasmic PTEN and by increasing Rak/PTEN association. One question remains, how is PTEN activated and then translocate to the plasma membrane? One possibility is that PTEN is the phosphatase that auto-dephosphorylates itself [68]. An important finding from the present study is that p17-mediated activation of PTEN is through a Tpr/p53-dependent manner. In addition to stabilizing PTEN, p17 is capable of activating PTEN by driving β-arrestin-mediated PTEN translocation from the cytoplasm to the plasma membrane via a Rock-1-dependent mechanism. Taken together, our results are suggestive of p17 acting as a positive regulator of PTEN.

Previous investigations have suggested that the PI3K/AKT pathway is activated in many virus infections, and some specific viral proteins have been demonstrated to enhance PI3K/AKT signaling via interaction with components of the pathway [69–73]. In this study, we found that p17 shuttles to the nucleus and exerts its suppressive effect on Tpr and nuclear signaling pathways. This may explain why ARV, a cytoplasmic virus, has a nucleocytoplasmic shuttling protein. In contrast to other viral products that have the potential to upregulate PI3K/AKT signaling [69–73], p17 inhibits PI3K/AKT/mTOR signaling via suppression of Tpr and activation of p53 and PTEN to create a better environment for its own replication. Furthermore, the RTK/PI3K/AKT pathway is one the most potent driving forces promoting tumor progression. Currently, targeting the deregulated PTEN/PI3K/AKT signaling axis has emerged as one of the major foci in anticancer drug development. In light of p17-mediated suppression of Tpr identified here, p17 serves a regulator of the p53/PTEN/AKT/mTOR and p53/p21 tumor suppressor-oncoprotein network. Targeting p53 or PTEN using p17 may be an effective strategy for the molecular therapy of human cancers.

The present study provides evidence demonstrating that p17 negatively controls mTORC1 signaling by up-regulating PTEN via a Tpr/p53/PTEN-dependent manner. Our previous and current studies together have demonstrated that p17 causes deactivation of eIF4E, eIF4G, and eIF4B [38, 39], thereby inhibiting protein synthesis downstream of mTORC1. Several recent reports have proved that activation of mTORC1 is required for cellular senescence [74, 75]. More rrecently, the choice between p53-induced senescence and quiescence has been elucidated [17]. Conversely, we provide first the evidence that suggests that p17 deactivates the mTOR pathway via suppression of Tpr and activates p53 and PTEN, implying that p17 might modulate cellular senescence by its ability to inhibit the mTORC1 pathway.

A variety of extracellular signal targets regulate cyclin D1-CDK4, which exert their critical functions during middle to late G1 phase by phosphorylating key substrates, including Rb (S780). Data obtained in this study suggests that p17 downregulates cyclin D1 in contrast to other cyclins. How does p17 downregulate cyclin D1? It has been suggested that transcription of the cyclin D1 gene, its synthesis and assembly with Cdk4, and the stability and nuclear retention of the holoenzyme rely strongly on receptor-mediated Ras and PI3K signaling [76]. Much evidence also supports the ideas that AKT controls cyclin D1 expression and the level of CDK inhibitors. Activation of Akt pathway is crucial for G1/S progression, and inhibition of Akt leads to G1 arrest in many cell types [77, 78]. In this work, we provide evidence demonstrating that p17 downregulates cyclin D1 expression, likely through at least two independent signaling pathways: PI3K/AKT and Tpr/p53/PTEN/ERK. In addition to ERK, cyclin D1 expression may also be regulated by Tpr/p53/PTEN-dependent inhibition of mTORC1 pathway. This notion is supported by several previous investigations that mTORC1 and its downstream effector eIF4E seem to control cyclin D levels through several mechanisms [79]. Our results reveal that p17-mediated effects on G1 phase of cell cycle seem to be a consequence of p17 effects on the activation of p53, PTEN and p21 as well as the inhibition of Tpr, AKT, and mTORC1.

Another important finding is that p21 regulation is mediated by the Tpr/p53/PTEN/AKT signaling pathway. In the present study, we show that p17-mediated p21 activation occurs through mechanisms involving inhibition of Tpr and AKT as well as activation of p53 and PTEN. Our results revealed that suppression of Tpr by p17 causes p21 (T145) to localize to the nucleus, which in turn activates p21 and inhibits cell cycle. Our earlier study reported that the level of the CDK inhibitors p15, p16, and p27 were not regulated by p17 [37], implying that p21 is a major CDK inhibitor modulated by p17, which then leads to Rb dephosphorylation and activation. Our results also revealed that after cells were infected with ARV for 3 hours, depletion of CDK4 increased virus production, suggesting that ARV benefits from G1 and G1/S cell cycle arrest. The cell cycle blockade enhances ARV replication by diverting the cellular machinery required for normal cell cycle to virus replication.

Our earlier study suggested that p17 induced autophagosome formation benefits virus replication by activating AMPK, p53/PTEN, and PKR/eIF2α signaling pathways [39]. This study further elucidates that p17-triggered autophagy via suppression of Tpr and activation of p53 and PTEN negatively regulates the function of mTORC1, thereby inducing autophagosome formation. The current and previous studies revealed that depletion of Beclin 1, Atg7, and LC3 in ARV-infected cells restricted virus replication [39], indicating that autophagy is important for virus replication. The different stages of autophagy might play divergent roles in virus life cycles. Formation of autophagosomes has been proven to offer a platform for viral replication and viral assembly [80, 81]. More recently, it was demonstrated that the lipids degraded in the late stage of autophagy were utilized as an energy source for dengue virus (DENV) replication [82]. In the case of ARV, the virus triggers the fusion of autophagosome with lysosome into autolysosome without completing the autophagy flux [39]. The precise mechanism by which p17-induced autophagosome formation benefits ARV replication remains to be explored.

**Fig 11:**
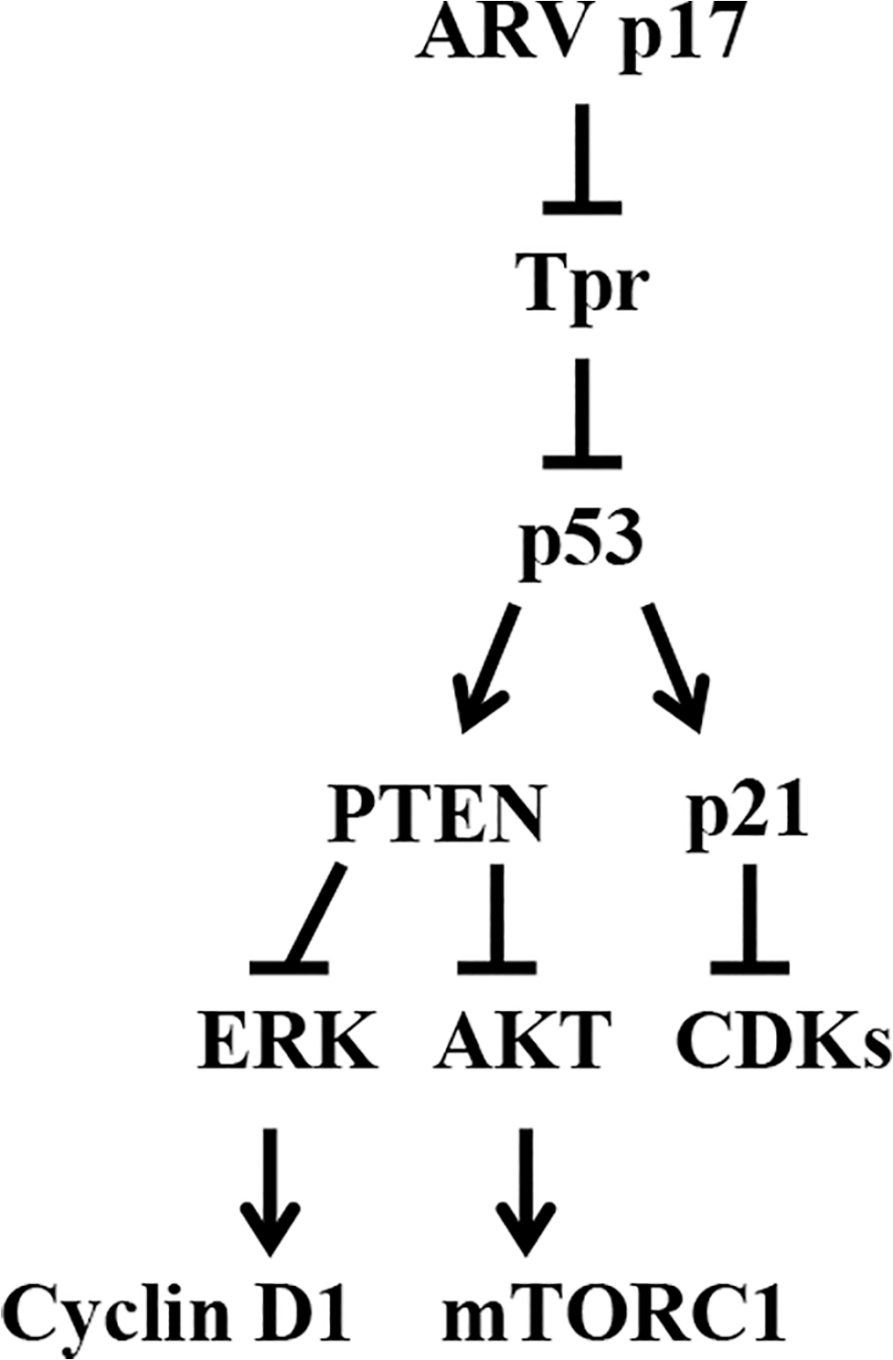
A model depicting the mechanisms of p17 modulating Tpr, p53, AKT, p21, PTEN, mTORC1 that govern cell cycle and autophagosome formation. This study establishes a new regulatory network of p17 linking Tpr, p53, p21, PTEN, mTORC1, and Rb. p17 suppresses Tpr leading to p53 and p21 nuclear accumulation, which in turn activates p53, p21, and PTEN. Furthermore, it also serves as a positive regulator of PTEN. Activation of PTEN leads to inhibition of ERK and AKT that result in mTORC1 inhibition as well as cyclin D1 and CDK 4 inhibition, leading to Rb activation. This study provides evidences demonstrating that p17 regulates cell cycle through Tpr/p53/PTEN/AKT and Tpr/p53/p21 signaling pathways. By suppressing Tpr, p17 is able to negatively regulate PI3K/AKT/mTORC1 and consequently induce cellular translation shutoff and autophagosome formation enhancing virus replication. →: activation;⊥: Inhibition.

Understanding the molecular basis for ARV interaction with host factors and for ARV-induced changes can shed light on normal cellular events and on the specific ways that ARV gains control over its hosts. A model in Fig 11 illustrates a novel regulatory network of p17. p17 negatively regulates Tpr leading to activation of p53, p21, PTEN, and Rb that are the major regulators of AKT, mTORC1, ERK, CDK4, and E2F-1. It is worth to noting that ARV has evolved mechanisms that alter the physiology of its host cells during infection to increase its replication and to block the host response to its infection in ways that are crucial for completing its life cycle.

## Supporting Information

S1 Fig p17 serves as a Tpr suppressor leading to p-p53 (S15) and p-p21(T145) nuclear accumulation in DF-1 cells. (A) The expression levels of Tpr and p53 in ARV-infected and pcDNA3.1-p17-transfected DF-1cells were examined. Whole cell lysates and nuclear extracts were collected at either 24 hours postinfection or 24 hours post-transfection for Western blot assay. The p17 and Tpr levels in ARV-infected and pcDNA3.1-p17- transfected cells were analyzed by Western blot assay and compared to the negative controls (cell alone and vector only). (B) To examine whether the Tpr transcription is downregulated by p17, the Tpr mRNA level in ARV-infected and pcDNA3.1-p17- transfected DF-1 cells were compared to mock controls (cell alone and vector only). In semi-quantitative RT-PCR amplification of p17 and Tpr genes, DF-1 cells were transfected with pcDNA3.1-p17 or infected with ARV at an MOI of 10. The p17-transfected or ARV-infected cells were collected at 24 hours postinfection (hpi), and total RNAs were extracted for semi-quantitative RT-PCR. After electrophoretic separation in an agarose gel, PCR products were stained with ethidium bromide. Graph shown represents the mean± SD calculated from three independent experiments. (C) The levels of p-ATM and p-p53 (Ser 15) were examined in caffeine-treated vero cells. Cells were pretreated with caffeine for 1 hour and then either transfected with p17 or infected with ARV at an MOI of 10 for 18 hours. Whole cell lysates were collected at either 18 hpi or 18 hours post-transfection for Western blot assay. (D) To study whether Tpr depletion affects p53, p21, and PTEN nuclear accumulation in DF-1 cells, nuclear extracts from ARV-infected and p17-transfected cells were collected for Western blot assays. DF-1 cells were transfected with Tpr shRNA for 6 hours before being infected with ARV at an MOI of 10 for 18 hours. In a parallel experiment, DF-1 cells were co-transfected with pcDNA3.1-p17 and Tpr shRNA plasmid for 24 hours. Nuclear extracts were collected for Western blot assays using the indicated antibodies. Results were obtained from three independent experiments. The protein levels were normalized to those for β-actin or Histone H2A. The activation and inactivation folds indicated below each lane were normalized against those at mock controls (cell alone). The levels of indicated protein in the mock control (cell alone) were considered 1-fold.
(TIF)

S2 Fig p17 positively regulates PTEN and Rak expression levels and drives PTEN translocation from the cytoplasm to the plasma membrane (A) The levels of p17, p-p53, p-PTEN, Rak, and NEDD4-1 in pcDNA3.1-p17 and pcDNA3.1 (vector only) were examined by Western blot assay. Whole cell lysates were collected at the indicated time points, and the protein level was examined by Western blot assay with the indicated antibodies. (B) In the presence and absence of Y-27632 and TBB, the levels of p17, p-PTEN, cytoplasmic PTEN, plasma membrane-associated PTEN, cytoplasmic β-arrestin, plasma membrane-associated β-arrestin, and Rock-1 were examined in p17-transfected DF-1 cells and negative control (cell alone). DF-1 cells were pretreated with either Y-27632 (10 μM) or TBB (5 μM) for 2 hours, followed by transfection with pcDNA3.1-p17 and then incubated for 24 hours at 37°C. Both β-actin and Na+/K+ ATPase were used as loading controls. Graph on right panel shows the relative level of PTEN and β-arrestin in membrane in p17-transfected cells in the presence of Y-27632 or TBB versus cell alone. The protein levels were normalized to those for β-actin or Na+/K+ ATPase. The activation and inactivation folds indicated below each lane were normalized against those at 0 h. The levels of indicated proteins at 0 h were considered 1-fold. Results were obtained from three independent experiments.
(TIF)

S3 Fig p17 negatively regulates ERK, CDKs, and cyclin D1 and positively regulates Rb. (A) The p-p53, PTEN, p-ERK, and cyclin D1 levels in the cytoplasm and the nucleus were examined. Whole cell lysates were collected at the indicated time points, and the protein level was examined by Western blot assay with the indicated antibodies. (B) The levels of p-p21, CDK4, p-Rb, and E2F-1 in pcDNA3.1- p17- and pcDNA3.1 (vector only)-transfected DF-1 cells were examined by Western blot assay at the indicated time points. Phosphorylation and protein levels were determined by immunoblotting with the indicated antibodies. Results were obtained from three independent experiments. The protein levels were normalized to those for β-actin or Histone H2A. The activation and inactivation folds indicated below each lane were normalized against those at 0 h. The levels of indicated proteins at 0 h were considered 1-fold. Results were obtained from three independent experiments.
(TIF)

S4 Fig p17 downregulates Akt and its downstream molecules. The levels of AKT and its downstream molecules in pcDNA3.1-p17 or pcDNA3.1 (vector only)-transfected DF-1 cells were examined. Whole cell lysates were collected at the indicated time points, and the protein level was examined by Western blot assay with the indicated antibodies. The protein levels were normalized to those for β-actin. The activation and inactivation folds indicated below each lane were normalized against those at 0 h. The levels of indicated proteins at 0 h were considered 1-fold. Similar results were obtained from three independent experiments.
(TIF)

S5 Fig p17-mediated inactivation of AKT/mTORC1 signaling pathway occurs through activation PTEN. shRNA-mediated blockade of PTEN was performed. Vero and DF-1 cells were co-transfected with pcDNA3.1-p17 and PTEN shRNAs for 24 hours followed by Western blot analysis with indicated antibodies. In the negative controls, cells were also co-transfected with p17 and respective negative controls (pGFP-V-RS and Scramble shRNA plasmids) for 24 hours. Phosphorylation and protein levels were determined by immunoblotting with the indicated antibodies. The protein levels were normalized to those for β-actin. The levels of indicated proteins in the mock controls (cell only) were considered 1-fold.
(TIF)

S6 Fig Original images of blots with molecular weight (KDa). (TIF)

S7 Fig Original images of blots with molecular weight (KDa). (TIF)

S8 Fig Original images of blots with molecular weight (KDa). (TIF)

S1 Table The level of host factors and phosphorylated proteins in p17-transfected Vero cells at 24 hours posttransfection in compared to the mock control (vector only). (DOCX)
