# Correction: Avian Reovirus Protein p17 Functions as a Nucleoporin Tpr Suppressor Leading to Activation of p53, p21 and PTEN and Inactivation of PI3K/AKT/mTOR and ERK Signaling Pathways

**DOI:** 10.1371/journal.pone.0138627

**Published:** 2015-09-14

**Authors:** Wei-Ru Huang, Hung-Chuan Chiu, Tsai-Ling Liao, Kuo-Pin Chuang, Wing-Ling Shih, Hung-Jen Liu

The following information is missing from the Funding section: This project was supported by the Taichung Veterans General Hospital and National Chung Hsing University (TCVGH-NCHU1047602).

Two references are omitted from the first sentence of the “p17 Drives β-Arrestin-Mediated PTEN Translocation from the Cytoplasm to the Plasma Membrane via a Rock-1 Dependent Manner” subsection of the Results.

The sentence should read: It is well-known that the small GTPase, RhoA, was one of the known proteins to increase PTEN lipid phosphatase activity via its downstream effector Rho kinase (ROCK) [51, 52, Lima-Fernandes et al., 2011, Li et al., 2005].

The references are: Lima-Fernandes E, Enslen H, Camand E, Kotelevets L, Boularan C, Achour L, et al. (2011) Distinct functional outputs of PTEN signalling are controlled by dynamic association with β-arrestins. EMBO J. 30(13):2557–68.

Li Z, Dong X, Wang Z, Liu W, Deng N, Ding Y, et al. (2005) Regulation of PTEN by Rho small GTPases. Nat Cell Biol. 7(4):399–404.

Table 1 has been corrected for improved readability. Please see the corrected Table 1 here.

**Table:** 

Gene	Accession number	Sequence (5′-3′)*	Location	Expected size (bp)
**Primers for cloning**				
p17 (Flag-tagged, full length)	AF330703	F: CGGAATTCATGCAATGGCTCCGCCATACGA (EcoR I)	293–314	441
		R: GCTCTAGATCATAGATCGGCGTCAAATCGC (Xba I)	733–712	
p17 (His-tagged, full length)	AF330703	F: CCCAAGCTTATGCAATGGCTCCGCCATACGA (Hind III)	293–314	441
		R: CCGCTCGAGTCATAGATCGGCGTCAAATCGC (Xho I)	733–712	
p17 (27–146)	AF330703	F: CGGAATTCACAATGGCATCATTTACTGCTATAAC (EcoR I)	371–390	363
		R: AACTCGAGTCATAGATCGGCGTCAAATCGCC (Xho I)	733–711	
p17 (1–118)	AF330703	F: CGGAATTCACAATGCAATGGCTCCGCCATACG (EcoR I)	293–313	354
		R: AAACTCGAGTCAGGATTGAGACCCGCCATCCCAATG (Xho I)	646–623	
p17 (119–146)	AF330703	F: CGGAATTCACAATGATCGCAGCGAAGAGAGGTCGTC (EcoR I)	647–668	87
		R: AACTCGAGTCATAGATCGGCGTCAAATCGCC (Xho I)	733–711	
**Primers for semi-quantitative RT-PCR**				
p17 (Full length)	AF330703	F: ATGCAATGGCTCCGCCATACGA	293–314	441
		R: TCATAGATCGGCGTCAAATCGC	733–712	
Tpr	NM_003292	F: CACTCTGATCTTGGCCAGCTTG	6637–6658	479
		R: CCACGGACACCTTGTCTCAATG	7115–7094	
p53	NM_001047151	F: CCCTTCCCAGAAAACCTACC	384–403	223
		R: CTCCGTCATGTGCTGTGACT	606–587	
PTEN	NM_001260965	F: CGAACTGGTGTAATGATAT	578–594	330
		R: CATGAACTTGTCTTCCCGT	907–889	
Rak	NM_002031	F: TGGTCAGTTCGGCGAAGTATGG	1173–1194	346
		R: GGCAGCCAGATCTCTGTGAATG	1518–1497	
GAPDH	NM_002046	F: CACCACCATGGAGAAGGCTGGGGCTCA	480–506	454
		R: GGCAGGTTTCTCCAGACGGCAGGTCAG	933–907	

F: forward; R: reverse;

* Underlines indicate the restriction sites designed in the indicated primers
