# Supplementary figures and images for: Avian Reovirus Protein p17 Functions as a Nucleoporin Tpr Suppressor Leading to Activation of p53, p21 and PTEN and Inactivation of PI3K/AKT/mTOR and ERK Signaling Pathways
Source: PLoS One. 2015 Aug 5;10(8):e0133699. doi: 10.1371/journal.pone.0133699 (PMC4526660; doi:10.1371/journal.pone.0133699)

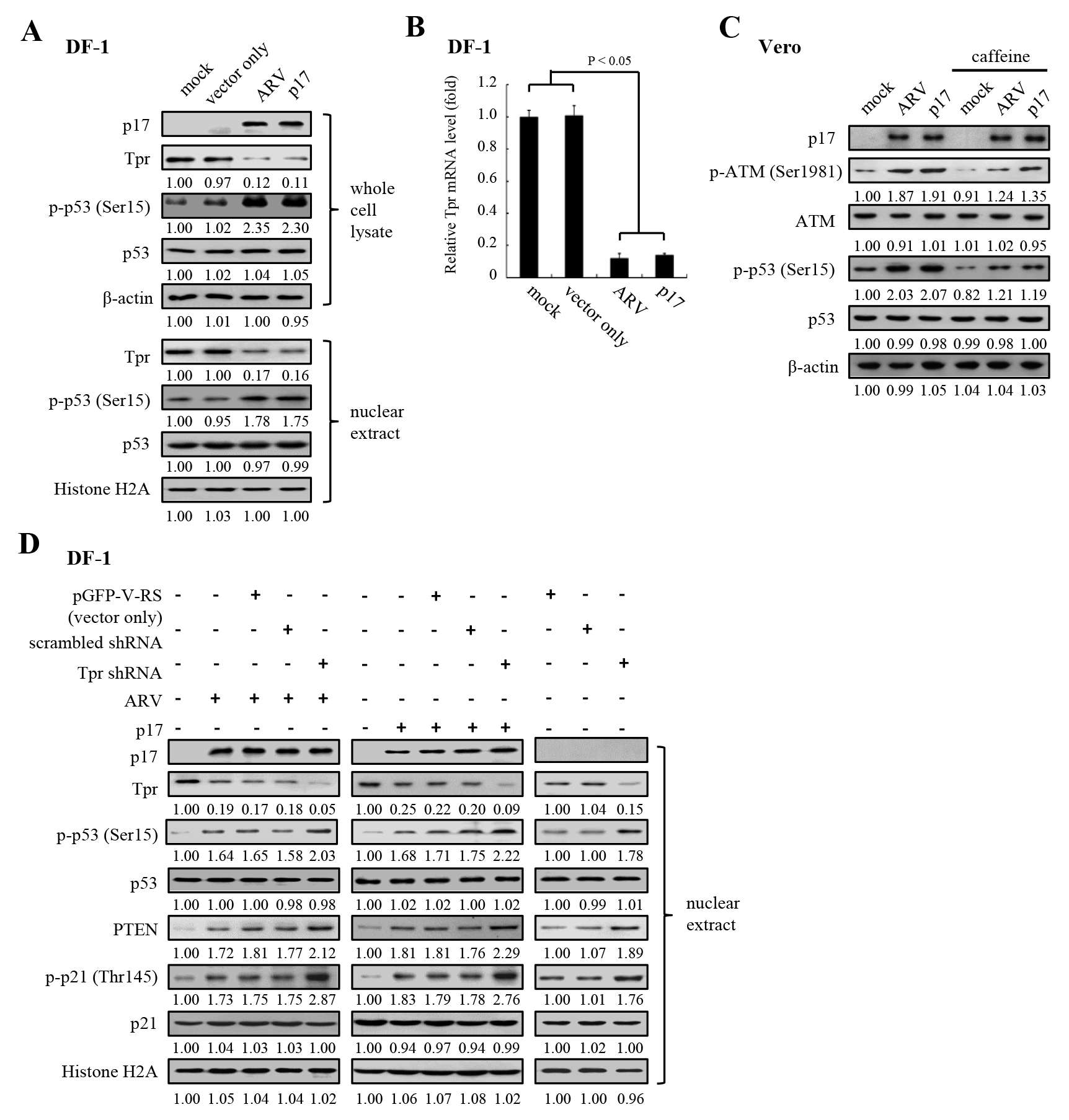

Supplement: S1 Fig — (A) The expression levels of Tpr and p53 in ARV-infected and pcDNA3.1-p17-transfected DF-1cells were examined. Whole cell lysates and nuclear extracts were collected at either 24 hours postinfection or 24 hours post-transfection for Western blot assay. The p17 and Tpr levels in ARV-infected and pcDNA3.1-p17- transfected cells were analyzed by Western blot assay and compared to the negative controls (cell alone and vector only). (B) To examine whether the Tpr transcription is downregulated by p17, the Tpr mRNA level in ARV-infected and pcDNA3.1-p17- transfected DF-1 cells were compared to mock controls (cell alone and vector only). In semi-quantitative RT-PCR amplification of p17 and Tpr genes, DF-1 cells were transfected with pcDNA3.1-p17 or infected with ARV at an MOI of 10. The p17-transfected or ARV-infected cells were collected at 24 hours postinfection (hpi), and total RNAs were extracted for semi-quantitative RT-PCR. After electrophoretic separation in an agarose gel, PCR products were stained with ethidium bromide. Graph shown represents the mean± SD calculated from three independent experiments. (C) The levels of p-ATM and p-p53 (Ser 15) were examined in caffeine-treated vero cells. Cells were pretreated with caffeine for 1 hour and then either transfected with p17 or infected with ARV at an MOI of 10 for 18 hours. Whole cell lysates were collected at either 18 hpi or 18 hours post-transfection for Western blot assay. (D) To study whether Tpr depletion affects p53, p21, and PTEN nuclear accumulation in DF-1 cells, nuclear extracts from ARV-infected and p17-transfected cells were collected for Western blot assays. DF-1 cells were transfected with Tpr shRNA for 6 hours before being infected with ARV at an MOI of 10 for 18 hours. In a parallel experiment, DF-1 cells were co-transfected with pcDNA3.1-p17 and Tpr shRNA plasmid for 24 hours. Nuclear extracts were collected for Western blot assays using the indicated antibodies. Results were obtained from three ind [file pone.0133699.s001.tif]

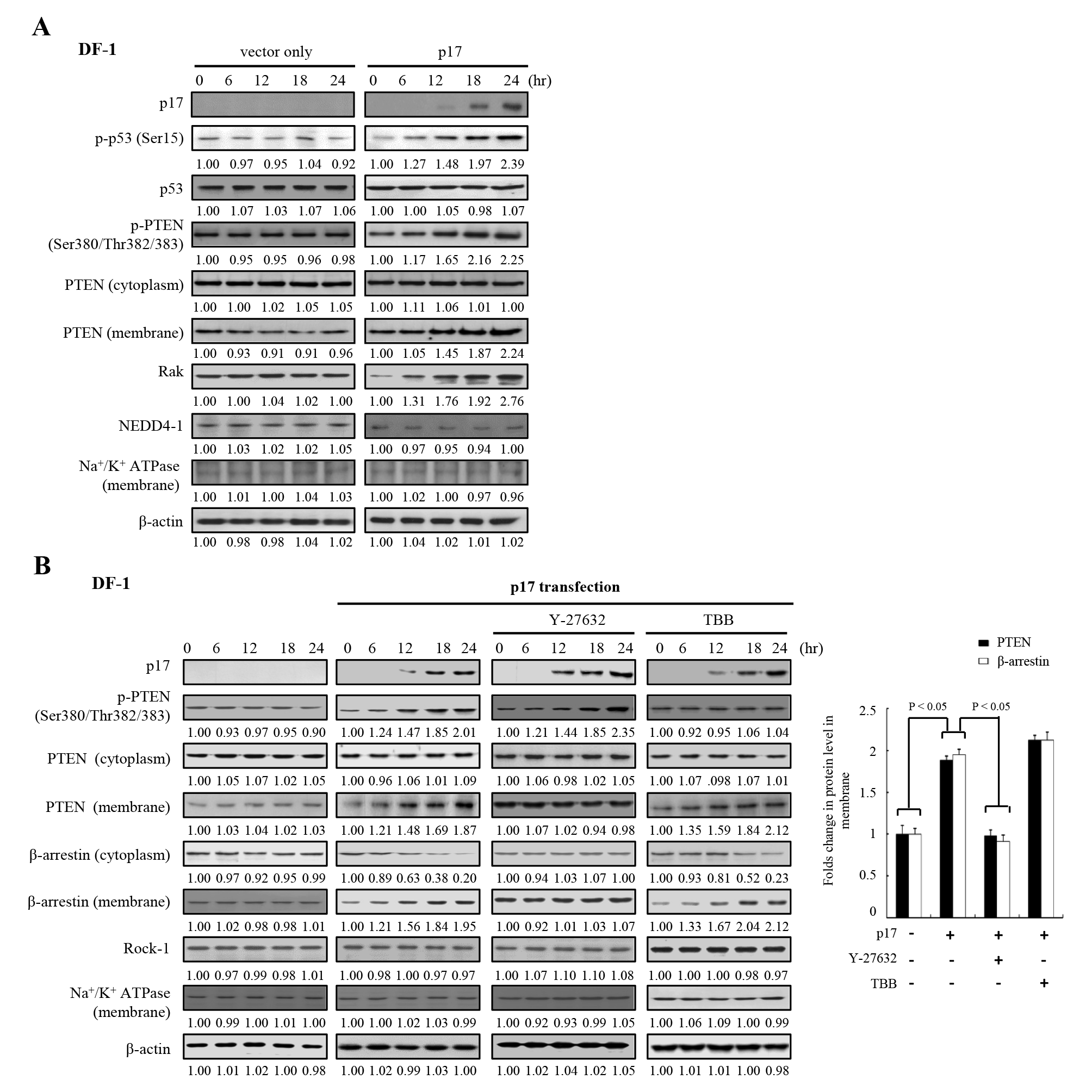

Supplement: S2 Fig — Whole cell lysates were collected at the indicated time points, and the protein level was examined by Western blot assay with the indicated antibodies. (B) In the presence and absence of Y-27632 and TBB, the levels of p17, p-PTEN, cytoplasmic PTEN, plasma membrane-associated PTEN, cytoplasmic β-arrestin, plasma membrane-associated β-arrestin, and Rock-1 were examined in p17-transfected DF-1 cells and negative control (cell alone). DF-1 cells were pretreated with either Y-27632 (10 μM) or TBB (5 μM) for 2 hours, followed by transfection with pcDNA3.1-p17 and then incubated for 24 hours at 37°C. Both β-actin and Na+/K+ ATPase were used as loading controls. Graph on right panel shows the relative level of PTEN and β-arrestin in membrane in p17-transfected cells in the presence of Y-27632 or TBB versus cell alone. The protein levels were normalized to those for β-actin or Na+/K+ ATPase. The activation and inactivation folds indicated below each lane were normalized against those at 0 h. The levels of indicated proteins at 0 h were considered 1-fold. Results were obtained from three independent experiments. (TIF) [file pone.0133699.s002.tif]

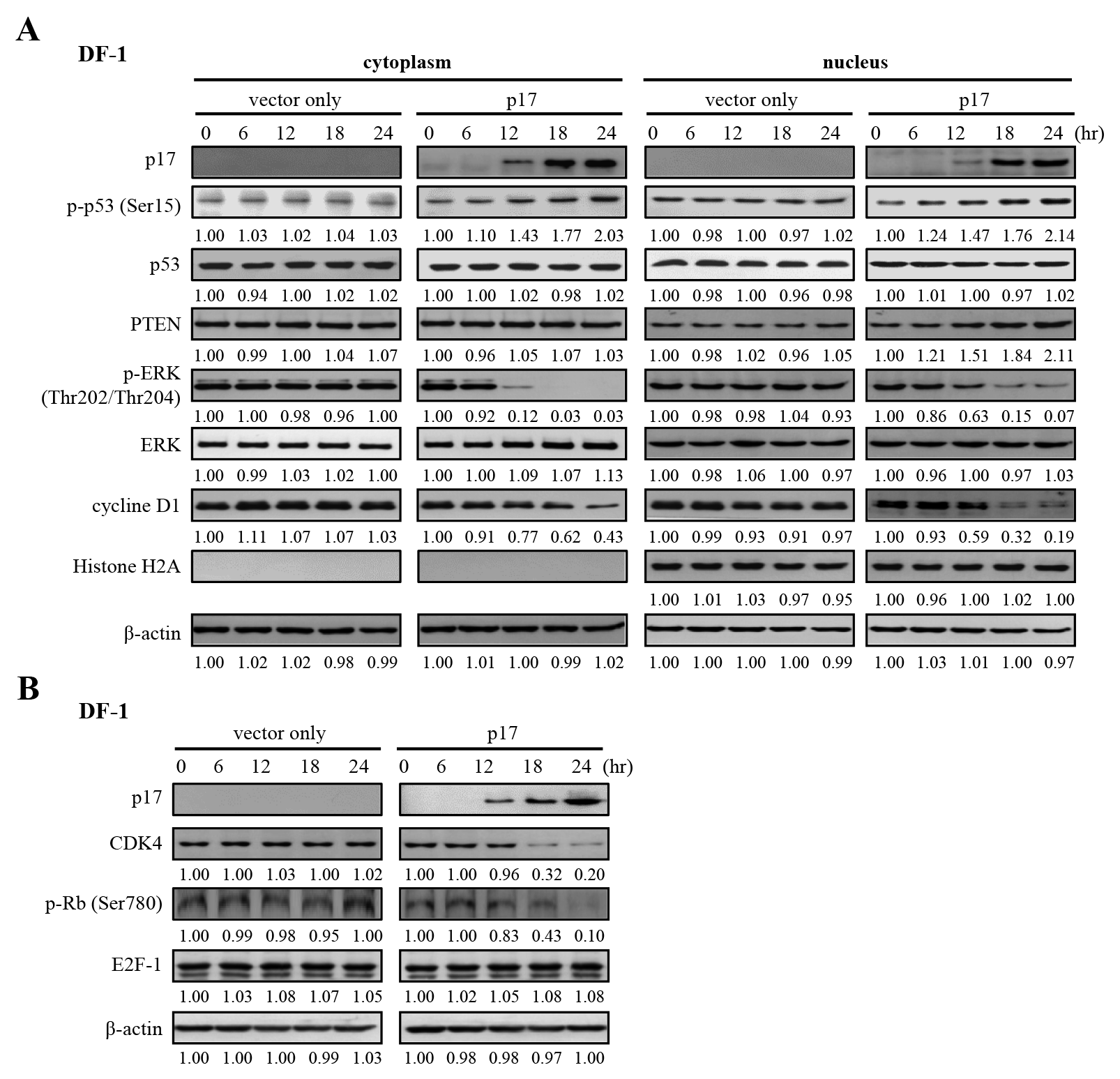

Supplement: S3 Fig — (A) The p-p53, PTEN, p-ERK, and cyclin D1 levels in the cytoplasm and the nucleus were examined. Whole cell lysates were collected at the indicated time points, and the protein level was examined by Western blot assay with the indicated antibodies. (B) The levels of p-p21, CDK4, p-Rb, and E2F-1 in pcDNA3.1- p17- and pcDNA3.1 (vector only)-transfected DF-1 cells were examined by Western blot assay at the indicated time points. Phosphorylation and protein levels were determined by immunoblotting with the indicated antibodies. Results were obtained from three independent experiments. The protein levels were normalized to those for β-actin or Histone H2A. The activation and inactivation folds indicated below each lane were normalized against those at 0 h. The levels of indicated proteins at 0 h were considered 1-fold. Results were obtained from three independent experiments. (TIF) [file pone.0133699.s003.tif]

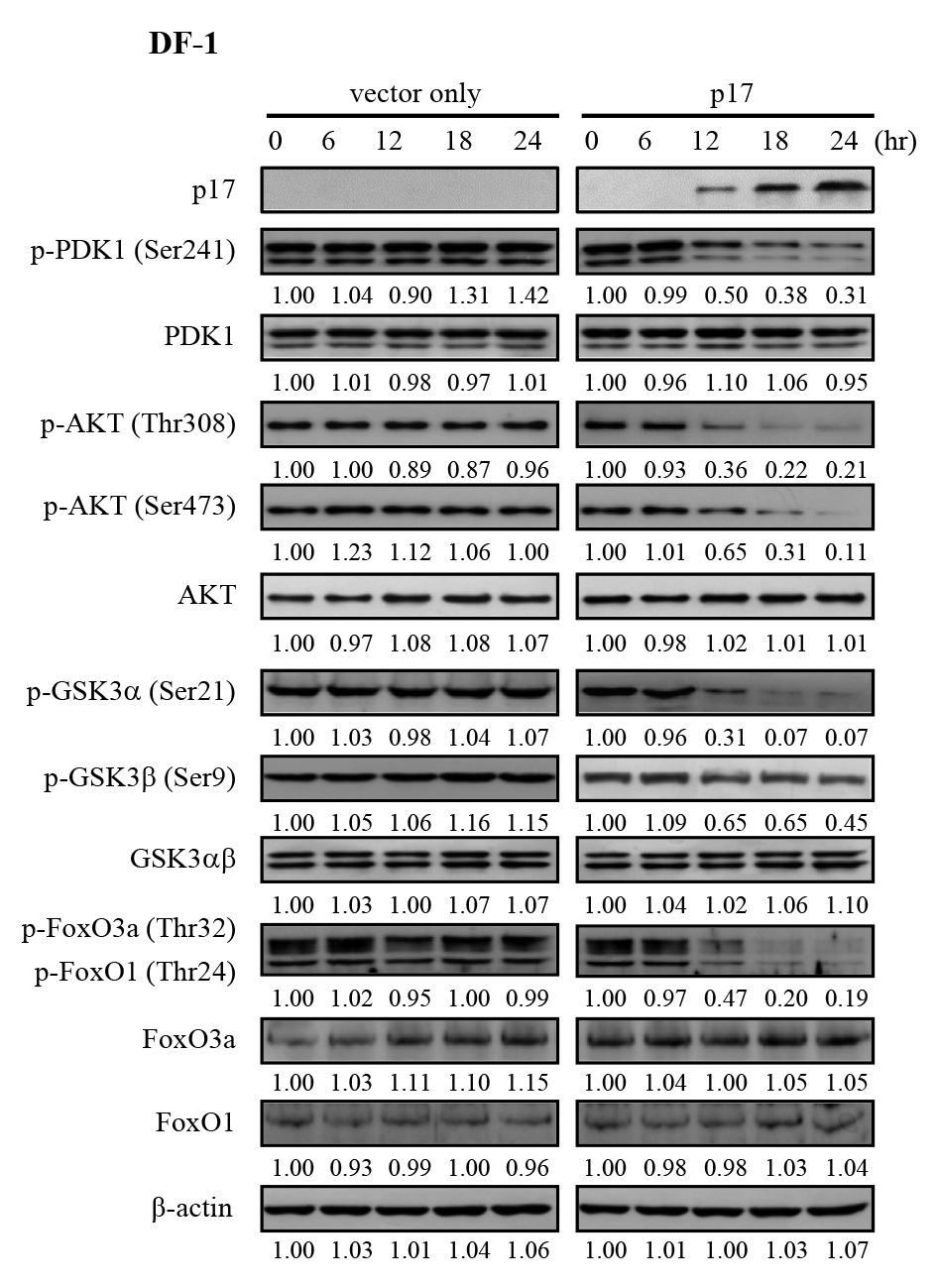

Supplement: S4 Fig — The levels of AKT and its downstream molecules in pcDNA3.1-p17 or pcDNA3.1 (vector only)-transfected DF-1 cells were examined. Whole cell lysates were collected at the indicated time points, and the protein level was examined by Western blot assay with the indicated antibodies. The protein levels were normalized to those for β-actin. The activation and inactivation folds indicated below each lane were normalized against those at 0 h. The levels of indicated proteins at 0 h were considered 1-fold. Similar results were obtained from three independent experiments. (TIF) [file pone.0133699.s004.tif]

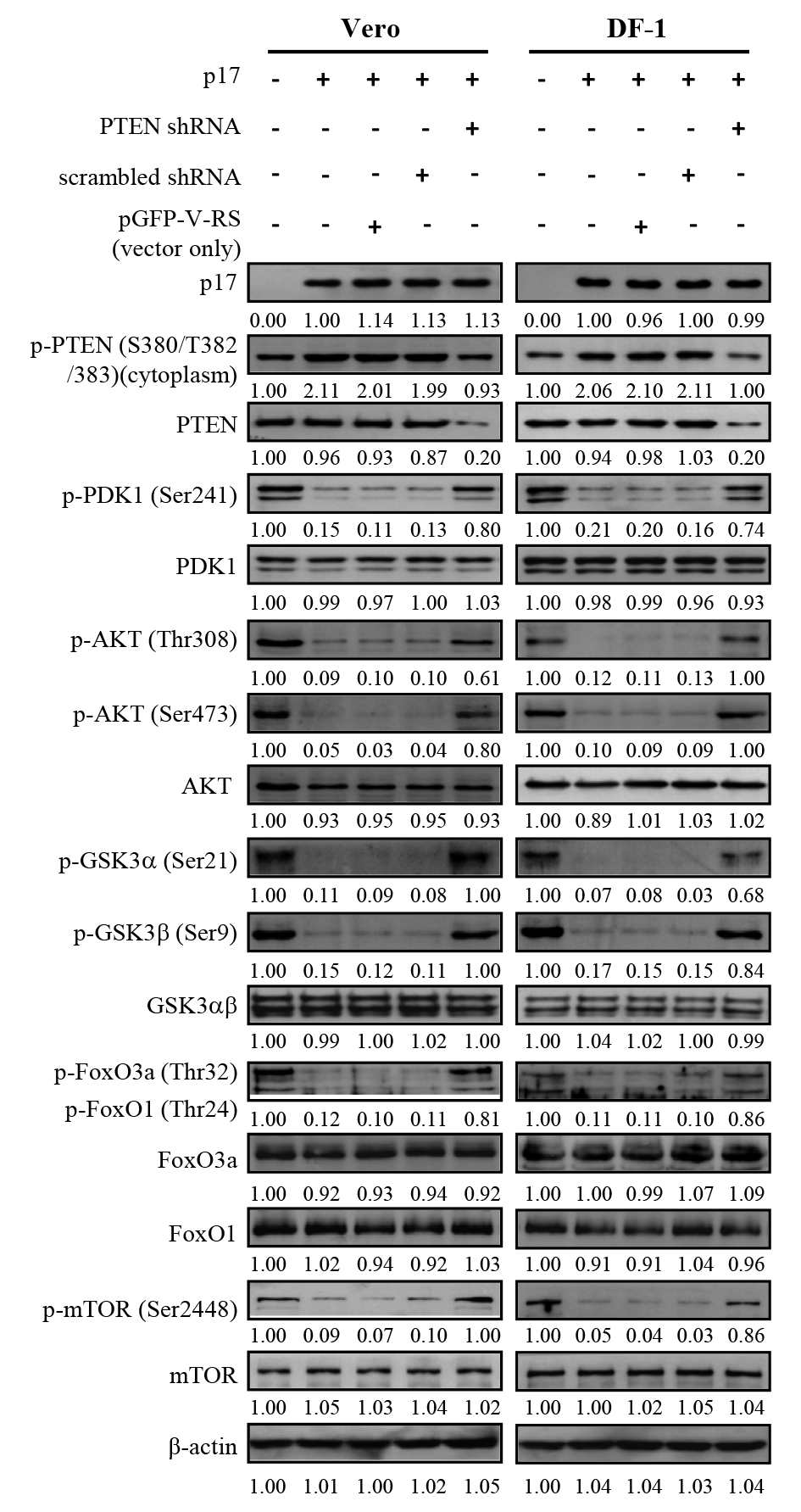

Supplement: S5 Fig — shRNA-mediated blockade of PTEN was performed. Vero and DF-1 cells were co-transfected with pcDNA3.1-p17 and PTEN shRNAs for 24 hours followed by Western blot analysis with indicated antibodies. In the negative controls, cells were also co-transfected with p17 and respective negative controls (pGFP-V-RS and Scramble shRNA plasmids) for 24 hours. Phosphorylation and protein levels were determined by immunoblotting with the indicated antibodies. The protein levels were normalized to those for β-actin. The levels of indicated proteins in the mock controls (cell only) were considered 1-fold. (TIF) [file pone.0133699.s005.tif]

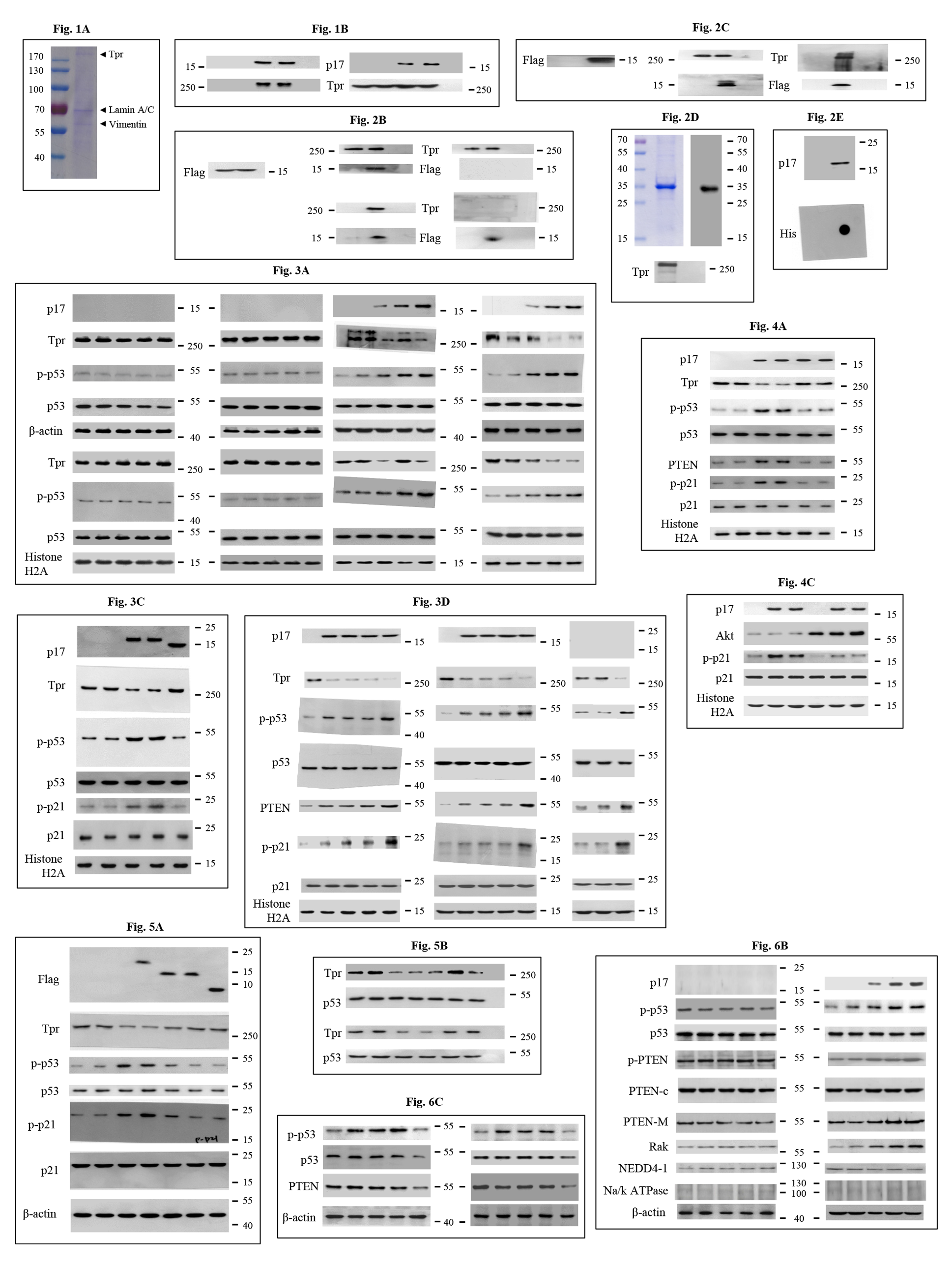

Supplement: S6 Fig — (TIF) [file pone.0133699.s006.tif]

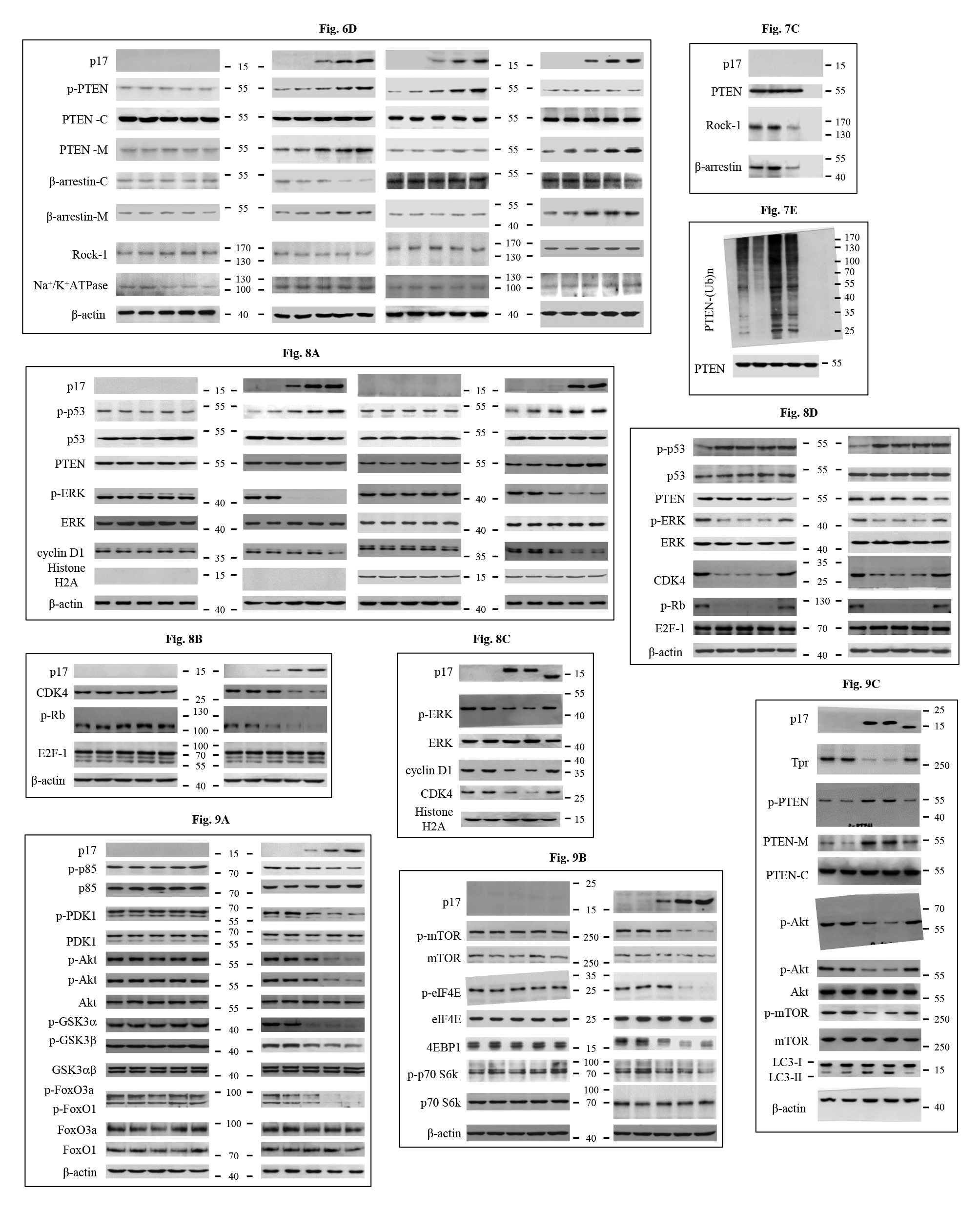

Supplement: S7 Fig — (TIF) [file pone.0133699.s007.tif]

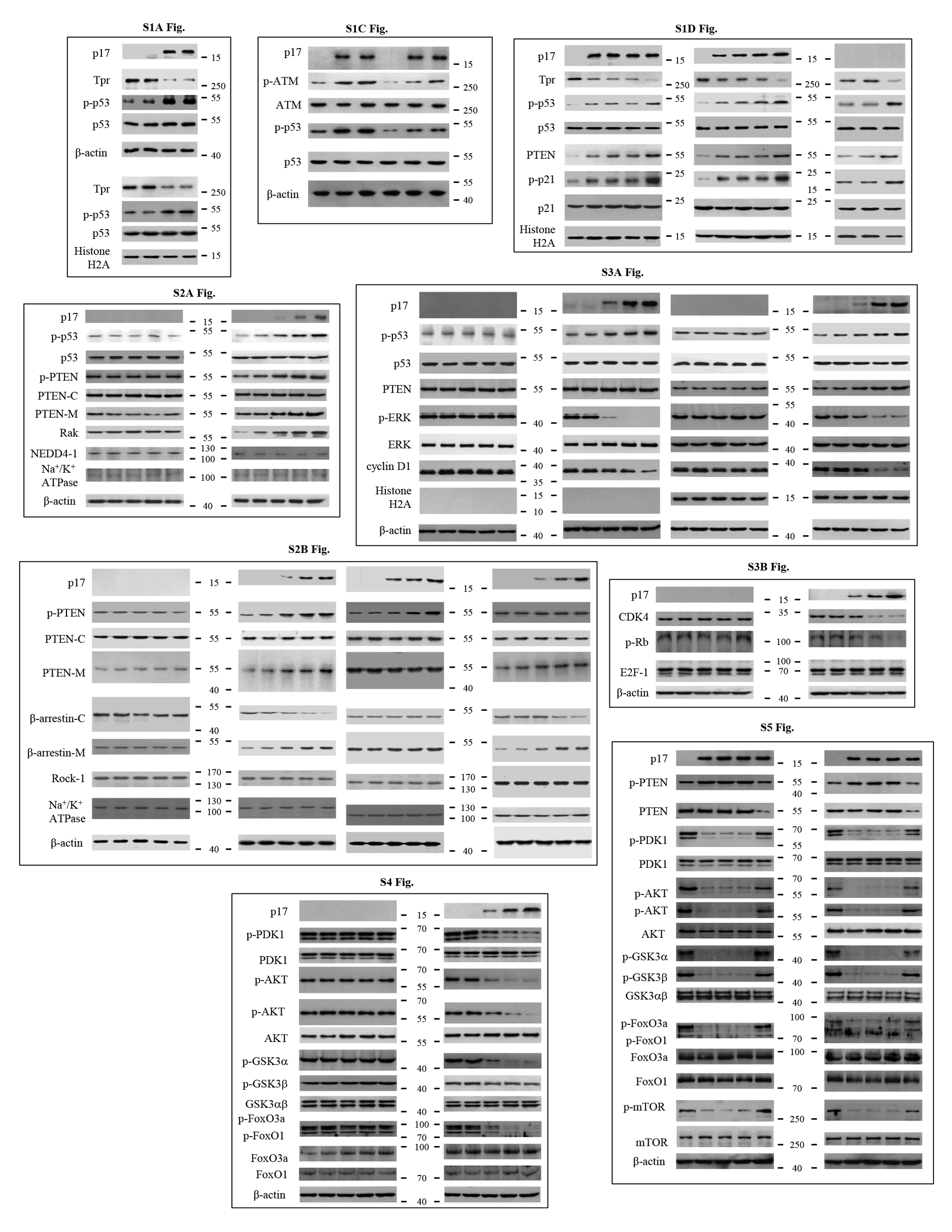

Supplement: S8 Fig — (TIF) [file pone.0133699.s008.tif]
