# Supplementary material for: Avian Reovirus Protein p17 Functions as a Nucleoporin Tpr Suppressor Leading to Activation of p53, p21 and PTEN and Inactivation of PI3K/AKT/mTOR and ERK Signaling Pathways
Source: PLoS One. 2015 Aug 5;10(8):e0133699. doi: 10.1371/journal.pone.0133699 (PMC4526660; doi:10.1371/journal.pone.0133699)
Supplement: S1 Table — (DOCX) [file pone.0133699.s009.docx]

S1 Table. The level of host factors and phosphorylated proteins in p17-transfected Vero cells at 24 hours posttransfection in compared to the mock control (vector only)

| Host factors/ phosphorylated proteins | Up or down regulation (folds) | | Host factors/ phosphorylated proteins | Up or down regulation (folds) | |
| --- | --- | --- | --- | --- | --- |
| Nuclear pore complex (NPC) | | | p-FoxO3a (Thr32) | | -- |
| Tpr | | --- ^b^ | p-FoxO1 (Thr24) | | -- |
| PTEN and its regulated proteins | | | mTORC1 | |  |
| PTEN (membrane) | | + ^a^ | p-mTOR (Ser2448) | | -- |
| PTEN (cytoplasm) | | c | p-eIF4E (Ser209) | | -- |
| p-PTEN (Ser380/ Thr382/383) | | + | p-p70 S6K (Thr389) | | -- |
| β-arrestin (membrane) | | + | 4E-BP1 | | -- |
| β-arrestin (cytoplasm) | | -- | Autophagosome formation | |  |
| Rak | | + | Beclin-1 | | ++ |
| Rock-1 | | c | LC3-II | | + |
| NEDD4-1 | | c | Cell cycle | |  |
| PI3k-Akt pathway | | | p-p53 (Ser15)(nucleus) | | + |
| p-p85 (Tyr458) | | - | p-p21 (Thr145)(nucleus) | | + |
| p-PDK-1 (Ser241) | | - | p-ERK (Thr202/Thr204)(nucleus) | | --- |
| p-Akt (Thr308) | | -- | cycline D1 (nucleus) | | -- |
| p-Akt (Ser473) | | -- | CDK4 | | -- |
| p-GSK3α (Ser21) | | --- | p-Rb (Ser780) | | --- |
| p-GSK3β (Ser9) | | -- | E2F-1 | | c |

^a^ Increase (+ ; 2 to 3.5-fold, ++ ; >3.5 to 7-fold); ^b^Decrease (- ; 2 to 3.5-fold, -- ; >3.5 to 7-fold, --- ; >7-fold); ^c^No significant changes; data shown represent the mean ±SD calculated from three independent experiments
